# When do microcircuits produce beyond-pairwise correlations?

**DOI:** 10.3389/fncom.2014.00010

**Published:** 2014-02-06

**Authors:** Andrea K. Barreiro, Julijana Gjorgjieva, Fred Rieke, Eric Shea-Brown

**Affiliations:** ^1^Department of Applied Mathematics, University of WashingtonSeattle, WA, USA; ^2^Department of Applied Mathematics and Theoretical Physics, University of CambridgeCambridge, UK; ^3^Department of Physiology and Biophysics, University of WashingtonSeattle, WA, USA

**Keywords:** retinal ganglion cells, maximum entropy distribution, stimulus-driven, correlations, computational model

## Abstract

Describing the collective activity of neural populations is a daunting task. Recent empirical studies in retina, however, suggest a vast simplification in how multi-neuron spiking occurs: the activity patterns of retinal ganglion cell (RGC) populations under some conditions are nearly completely captured by pairwise interactions among neurons. In other circumstances, higher-order statistics are required and appear to be shaped by input statistics and intrinsic circuit mechanisms. Here, we study the emergence of higher-order interactions in a model of the RGC circuit in which correlations are generated by common input. We quantify the impact of higher-order interactions by comparing the responses of mechanistic circuit models vs. “null” descriptions in which all higher-than-pairwise correlations have been accounted for by lower order statistics; these are known as pairwise maximum entropy (PME) models. We find that over a broad range of stimuli, output spiking patterns are surprisingly well captured by the pairwise model. To understand this finding, we study an analytically tractable simplification of the RGC model. We find that in the simplified model, bimodal input signals produce larger deviations from pairwise predictions than unimodal inputs. The characteristic light filtering properties of the upstream RGC circuitry suppress bimodality in light stimuli, thus removing a powerful source of higher-order interactions. This provides a novel explanation for the surprising empirical success of pairwise models.

## 1. Introduction

Information in neural circuits is often encoded in the activity of large, highly interconnected neural populations. The combinatoric explosion of possible responses of such circuits poses major conceptual, experimental, and computational challenges. How much of this potential complexity is realized? What do statistical regularities in population responses tell us about circuit architecture? Can simple circuit models with limited interactions among cells capture the relevant information content? These questions are central to our understanding of neural coding and decoding.

Two developments have advanced studies of synchronous activity in recent years. First, new experimental techniques provide access to responses from the large groups of neurons necessary to adequately sample synchronous activity patterns (Baudry and Taketani, 2006). Second, maximum entropy approaches from statistical physics have provided a powerful approach to distinguish genuine higher-order synchrony (correlations) from that explainable by pairwise statistical interactions among neurons (Martignon et al., 2000; Amari, 2001; Schneidman et al., 2003). These approaches have produced diverse findings. In some instances, activity of neural populations is extremely well described by pairwise interactions alone, so that pairwise maximum entropy (PME) models provide a nearly complete description (Shlens et al., 2006, 2009). In other cases, while pairwise models bring major improvements over independent descriptions, it is not clear that they fully capture the data (Martignon et al., 2000; Schneidman et al., 2006; Tang et al., 2008; Yu et al., 2008; Montani et al., 2009; Ohiorhenuan et al., 2010; Santos et al., 2010). Empirical studies indicate that pairwise models can fail to explain the responses of spatially localized triplets of cells (Ohiorhenuan et al., 2010; Ganmor et al., 2011), as well as the activity of populations of ~100 cells responding to natural stimuli (Ganmor et al., 2011). Overall, the diversity of empirical results highlights the need to understand the network and input features that control the statistical complexity of synchronous activity patterns.

Several themes have emerged from efforts to link the correlation structure of spiking activity to circuit mechanisms using both abstract (Amari et al., 2003; Krumin and Shoham, 2009; Macke et al., 2009; Roudi et al., 2009a) and biologically-based models (Bohte et al., 2000; Martignon et al., 2000; Roudi et al., 2009b); these models, however, do not provide a full description for why the PME models succeed or fail to capture neural circuit dynamics. First, thresholding non-linearities in circuits with Gaussian input signals can generate correlations that cannot be explained by pairwise statistics (Amari et al., 2003); the deviations from pairwise predictions are modest at moderate population sizes (Macke et al., 2009), but may become severe as population size grows large (Amari et al., 2003; Macke et al., 2011). The pairwise model also fails in networks of recurrent integrate-and-fire units with adapting thresholds and refractory potassium currents (Bohte et al., 2000). The same is true for “Boltzmann-type” networks with hidden units (Koster et al., 2013). Finally, small groups of model neurons that perform logical operations can be shown to generate higher-order interactions by introducing noisy processes with synergistic effects (Schneidman et al., 2003), but it is unclear what neural mechanisms might produce similar distributions. These diverse findings point to the important role that circuit features and mechanisms—input statistics, input/output relationships, and circuit connectivity—can play in regulating higher-order interactions. Nevertheless, we lack a systematic understanding that links these features and their combinations to the success and failure of pairwise statistical models.

A second theme that has emerged is the use of perturbation approaches to explain why maximum entropy models with purely pairwise interactions capture circuit behavior in the limit in which the population firing rate is very low (i.e., the total number of firing events from all cells in the same small time window is small) (Cocco et al., 2009; Roudi et al., 2009a; Tkacik et al., 2009). Also in this regime, higher-order interactions cannot be introduced as an artifact of under-sampling the network (Tkacik et al., 2009), a concern at higher population firing rates. However, the low to moderate population firing rates observed in many studies permit *a priori* a fairly broad range in the quality of pairwise fits. What is left to explain then is why circuits operating outside the low population firing rate regime often produce fits consistent with the PME model.

We approach this issue here by systematically characterizing the ability of PME models to capture the responses of a class of circuit models with the following defining features. First, we consider relatively small circuits of 3–16 cells, each with identical intrinsic dynamics (i.e., spike-generating mechanism and level of excitability). Second, we assume a particular structure for inputs across the circuit. Each neuron receives the same global input which, for example, represents stimuli in the receptive fields of all modeled cells. Neurons also receive an independent, Gaussian-like noise term. Third, the circuit has either no reciprocal coupling, or has all-to-all excitatory or gap junction coupling. We begin with circuit models fully constrained by measured properties of primate ON parasol ganglion networks, receiving full-field and checkerboard light inputs. We then explore a simple thresholding model for which we exhaustively search over the entire parameter space.

We identify general principles that describe higher-order spike correlations in the circuits we study. First, in all cases we examined, the overall strength of higher-order correlations are constrained to be far lower than the statistically possible limits. Second, for the higher-order correlations that do occur, the primary factor that determines how significant they will be is the bimodal vs. unimodal profile of the common input signal. A secondary factor is the strength of recurrent coupling, which has a non-monotonic impact on higher-order correlations. Our findings provide insight into why some previously measured activity patterns are well captured by PME descriptions, and provide predictions for the mechanisms that allow for higher-order spike correlations to emerge.

## 2. Results

### 2.1. Quantifying higher-order correlations in neural circuits

One strategy to identify higher-order interactions is to compare multi-neuron spike data against a description in which any higher-order interactions have been removed in a principled way—that is, a description in which all higher-order correlations are completely described by lower-order statistics. Such a description may be given by a maximum entropy model (Jaynes, 1957a,b; Amari, 2001), in which one identifies the most unstructured, or maximum entropy, distribution consistent with the constraints. Comparing the predicted and measured probabilities of different responses tests whether the constraints used are sufficient to explain observed network activity, or whether additional constraints need to be considered. Such constraints would produce additional structure in the predicted response distribution, and hence lower the entropy.

A common approach is to limit the constraints to a given statistical order—for example, to consider only the first and second moments of the distributions, which are determined by the mean and pairwise interactions. In the context of spiking neurons, we denote μ_*i*_ ≡ **E**[*x*_*i*_] as the firing rate of neuron *i* and $\hat{\rho }$_*ij*_ ≡ **E** [*x*_*i*_ *x*_*j*_] as the joint probability that neurons *i* and *j* will fire. The distribution with the largest entropy for a given μ_*i*_ and $\hat{\rho }$_*ij*_ is referred to as the *PME* model.

We use the Kullback–Leibler divergence, *D*_KL_(*P*, $\tilde{P}$), to quantify the accuracy of the PME approximation $\tilde{P}$ to a distribution *P*. This measure has a natural interpretation as the contribution of higher-order interactions to the response entropy *S*(*P*) (Amari, 2001; Schneidman et al., 2003), and may in this context be written as the difference of entropies *S*($\tilde{P}$) − *S*(*P*). In addition, *D*_KL_(*P*, $\tilde{P}$) is approximately −log_2_*L*, where *L* is the average likelihood (over different observations) that a sequence of data drawn from the distribution *P* was instead drawn from the model $\tilde{P}$ (Cover and Thomas, 1991; Shlens et al., 2006). For example, if *D*_KL_(*P*, $\tilde{P}$) = 1, the average likelihood that a single sample, i.e., a single network response, came from $\tilde{P}$ relative to the likelihood that it came from *P* is 2^−1^ (we use the base 2 logarithm in our definition of the Kullback–Leibler divergence, so all numerical values are in units of bits).

An alternative measure of the quality of the pairwise model comes from normalizing *D*_KL_(*P*, $\tilde{P}$) by the corresponding distance of the distribution *P* from an *independent maximum entropy* fit *D*_KL_(*P, P*_1_), where *P*_1_ is the highest entropy distribution consistent with the mean firing rates of the cells (equivalently, the product of single-cell marginal firing probabilities) (Amari, 2001). Many studies (Schneidman et al., 2006; Shlens et al., 2006, 2009; Roudi et al., 2009a) use

(1) $$\Delta =1-\frac{D_{\text{KL}}\left(P,\tilde{P}\right)}{D_{\text{KL}}\left(P,P_{1}\right)};$$

a value of Δ = 1 indicates that the pairwise model perfectly captures the additional information left out of the independent model, while a value of Δ = 0 indicates that the pairwise model gives no improvement over the independent model. To aid comparison with other studies, we report values of Δ in parallel with *D*_KL_(*P*, $\tilde{P}$) when appropriate.

We next explore and interpret the achievable range of *D*_KL_(*P*, $\tilde{P}$) values. The problem is made simpler if, following previous studies (Bohte et al., 2000; Amari, 2001; Macke et al., 2009; Montani et al., 2009), we consider only permutation-symmetric spiking patterns, in which the firing rate and correlation do not depend on the identity of the cells; i.e., μ_*i*_ = μ, $\hat{\rho }$_*ij*_ = $\hat{\rho }$ for *i* ≠ *j*. We start with three cells having binary responses and assume that the response is stationary and uncorrelated in time. From symmetry, the possible network responses are

$$\begin{array}{l} p_{0}=P\left[\left(0,0,0\right)\right]\\ p_{1}=P\left[\left(1,0,0\right)\right]=P\left[\left(0,1,0\right)\right]=P\left[\left(0,0,1\right)\right]\\ p_{2}=P\left[\left(1,1,0\right)\right]=P\left[\left(1,0,1\right)\right]=P\left[\left(0,1,1\right)\right]\\ p_{3}=P\left[\left(1,1,1\right)\right], \end{array}$$

where *p*_*i*_ denotes the probability that a particular set of *i* cells spike and the remaining 3 − *i* do not. Possible values of (*p*_0_, *p*_1_, *p*_2_, *p*_3_) are constrained by the fact that *P* is a probability distribution, so that the sum of *p*_*i*_ over all eight states is one.

**Figure 1:**
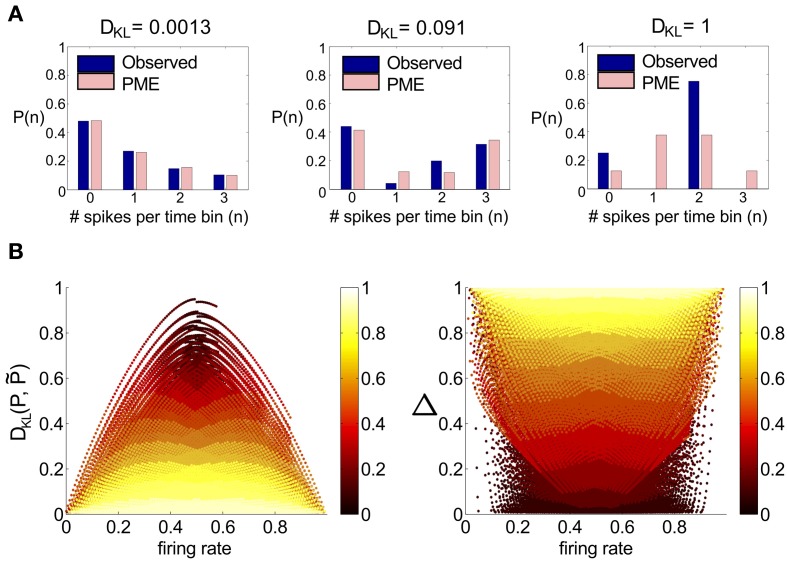
**A survey of the quality of the pairwise maximum entropy (PME) model for symmetric spiking distributions on three cells**. **(A)** Probability distribution *P* (dark blue) and pairwise approximation $\tilde{P}$ (light pink) for three example distributions. From left to right: an example from the simple sum-and-threshold model receiving skewed common input; an example from the sum-and-threshold model receiving bimodal common input [specifically, the distribution with maximal *D*_KL_(*P*, $\tilde{P}$)]; a specific probability distribution resulting from application of the XOR operator [for illustration of a “worst case” fit of the PME model (Schneidman et al., 2003)]. **(B)** *D*_KL_(*P*, $\tilde{P}$) vs. firing rate and Δ vs. firing rate, for a comprehensive survey of possible symmetric spiking distributions on three cells (see text for details). Firing rate is defined as the probability of a spike occurring per cell per random draw of the sum-and-threshold model, as defined in Equation (16). Color indicates output correlation coefficient ρ ranging from black for ρ ∈ (0, 0.1), to white for ρ ∈ (0.9, 1), as illustrated in the color bars.

To assess the numerical significance of *D*_KL_(*P*, $\tilde{P}$), we can compare it with the maximal achievable value for any symmetric distribution on three spiking cells. For three cells, the maximal value is *D*_KL_(*P*, $\tilde{P}$) = 1 (or 1/3 bits per neuron), achieved by the XOR operation (Schneidman et al., 2003). This distribution is illustrated in Figure 1A (right), together with two distributions produced by our mechanistic circuit models—illustrating observed deviations from PME fits for unimodal (left) and bimodal (middle) distributions of inputs (see below). The *KL*-divergence for these two patterns is 0.0013 and 0.091, respectively. As suggested by these bar plots (and explored in detail below), the distributions produced by a wide set of mechanistic circuit models are quite well captured by the PME approximation: to use the likelihood interpretation described above, an observer would need to draw many more samples from these distributions in order to distinguish between the true and model distributions: ≈1000 times and ≈10 times, respectively, in comparison to the XOR operator.

To further identify appropriate “benchmark” values of *D*_KL_(*P*, $\tilde{P}$) with which to compare our mechanistic circuit models, in Figure 1B we show plots of *D*_KL_(*P*, $\tilde{P}$) and Δ vs. firing rate produced by an exhaustive sampling of symmetric distributions on three cells. From this picture, we can see that it is possible to find symmetric, three-cell spiking distributions that are poorly fit by the pairwise model at a range of firing rates and pairwise correlations, with the largest values of *D*_KL_(*P*, $\tilde{P}$) found at low correlations (note that the XOR distribution has an average pairwise covariance of zero (i.e., **E**[*X*_1_ *X*_2_] = **E**[*X*_1_] **E**[*X*_2_])).

#### 2.1.1. A condition for higher-order correlations

Possible solutions to the symmetric PME problem take the form of exponential functions characterized by two parameters, λ_1_ and λ_2_, which serve as Lagrange multipliers for the constraints:

(2) $$\begin{array}{l} P\left[\left(x_{1},x_{2},x_{3}\right)\right]=\frac{1}{Z}\exp[\lambda_{1}\left(x_{1}+x_{2}+x_{3}\right)+\\ \;\lambda_{2}\left(x_{1}x_{2}+x_{2}x_{3}+x_{1}x_{3}\right)]. \end{array}$$

The factor *Z* normalizes *P* to be a probability distribution.

By combining individual probabilities of events as given by Equation (2) the following relationship must be satisfied by any symmetric PME solution:

(3) $$\frac{p_{3}}{p_{0}}={\left(\frac{p_{2}}{p_{1}}\right)}^{3}.$$

This is equivalent to the condition that the *strain* measure of Ohiorhenuan and Victor (2010) be zero (in particular, the strain is negative whenever *p*_3_/*p*_0_ − (*p*_2_/*p*_1_)^3^ < 0, a condition identified in Ohiorhenuan and Victor (2010) as corresponding to sparsity in the neural code).

For three-cell, symmetric networks, models that exactly satisfy Equation (3) will also be exactly described via PME. Moreover, note that probability models that meet this constraint fall on a surface in the space of (normalized) histograms, given by the probabilities *p*_*j*_. One can verify by straightforward calculations (see Appendix) that—given fixed lower order moments—*D*_KL_(*P*, $\tilde{P}$) is a convex function of the probabilities *p*_*j*_. This has interesting consequences for predicting when large vs. small values of *D*_KL_(*P*, $\tilde{P}$) will be found (see Appendix).

It is not necessary to assume permutation symmetry when deriving the PME fit $\tilde{P}$ to an observed distribution *P*, or in computing derived quantities such as *D*_KL_(*P*, $\tilde{P}$), and we do not do so in this study. However, most of the distributions we study are derived from mechanistic models that are themselves symmetric or near-symmetric. Therefore, we anticipate that the simplified calculations for permutation-symmetric distributions will yield analytical insight into our findings.

### 2.2. Mechanisms that impact beyond-pairwise correlations in triplets of on-parasol retinal ganglion cells

Having established the range of beyond-pairwise correlations that are possible statistically, we turn our focus to coding in retinal ganglion cell (RGC) populations, an area that has received a great deal of attention empirically. Specifically, PME approaches have been effective in capturing the activity of small RGC populations (Schneidman et al., 2006; Shlens et al., 2006, 2009). This success does not have an obvious anatomical correlate; there are multiple opportunities in the retinal circuitry for interactions among three or more ganglion cells. We explored circuits composed of three RGC cells with input statistics, recurrent connectivity and spike-generating mechanisms based directly on experiment. We based our model on ON parasol RGCs, one of the RGC types for which PME approaches have been applied extensively (Shlens et al., 2006, 2009). In addition, by examining how marginal input statistics are shaped by stimulus filtering, we also reveal the role that the specific filtering properties of ON parasol cells have in shaping higher-order interactions.

#### 2.2.1. RGC model

We modeled a single ON parasol RGC in two stages (for details see section 4). First, we characterized the light-dependent excitatory and inhibitory synaptic inputs to cell *k* (*g*^exc^_*k*_(*t*), *g*^inh^_*k*_(*t*)) in response to randomly fluctuating light inputs *s*_*k*_(*t*) via a linear-nonlinear model, e.g.,:

(4) $$g_{k}^{\text{exc}}(t)=N^{\text{exc}}\left[L^{\text{exc}}*s_{k}(t)+\eta_{k}^{\text{exc}}\right],$$

where *N*^exc^ is a static non-linearity, *L*^exc^ is a linear filter, and η^exc^_*k*_ is an effective input noise that captures variability in the response to repetitions of the same time-varying stimulus. These parameters were determined from fits to experimental data collected under conditions similar to those in which PME models have been tested empirically (Shlens et al., 2006, 2009; Trong and Rieke, 2008). The modeled excitatory and inhibitory conductances captured many of the statistical features of the real conductances, particularly the correlation time and skewness (data not shown).

Second, we used Equation (4) and an equivalent expression for *g*^inh^_*k*_(*t*) as inputs to an integrate-and-fire model incorporating a non-linear voltage and history-dependent term to account for refractory interactions between spikes (Badel et al., 2007, 2008). The voltage evolution equation was of the form

(5) $$\frac{dV}{dt}=F\left(V,t-t_{\text{last}}\right)+\frac{I_{\text{input}}(t)}{C},$$

where *F*(*V*, *t* − *t*_last_) was allowed to depend on the time of the last spike *t*_last_. Briefly, we obtained data from a dynamic clamp experiment (Sharpe et al., 1993; Murphy and Rieke, 2006) in which currents corresponding to *g*^exc^(*t*) and *g*^inh^(*t*) were injected into a cell and the resulting voltage response measured. The input current *I*_input_ injected during one time step was determined by scaling the excitatory and inhibitory conductances by driving forces based on the measured voltage in the previous time step; that is,

(6) $$I_{\text{input}}(t)=-g^{\text{exc}}(t)\left(V-V_{E}\right)-g^{\text{inh}}(t)\left(V-V_{I}\right),$$

We used this data to determine *F* and *C* using the procedure described in Badel et al. (2007); details, including values of all fitted parameters, are described in section 4. Recurrent connections were implemented by adding an input current proportional to the voltage difference between the two coupled cells.

The prescription above provided a flexible model that allowed us to study the responses of three-cell RGC networks to a wide range of light inputs and circuit connectivities. Specifically, we simulated RGC responses to light stimuli that were (1) constant, (2) time-varying and spatially uniform, and (3) varying in both space and time. Correlations between cell inputs arose from shared stimuli, from shared noise originating in the retinal circuitry (Trong and Rieke, 2008), or from recurrent connections (Dacey and Brace, 1992; Trong and Rieke, 2008). Shared stimuli were described by correlations among the light inputs *s*_*k*_. Shared noise arose via correlations in η^exc^_*k*_ and η^ink^_*k*_ as described in section 4. The recurrent connections were chosen to be consistent with observed gap-junctional coupling between ON parasol cells. We also investigated how stimulus filtering by *L*^exc^ and *L*^inh^ influenced network statistics. To compare our results with empirical studies, constant light, and spatially and temporally fluctuating checkerboard stimuli were used as in Shlens et al. (2006, 2009).

#### 2.2.2. The feedforward RGC circuit is well-described by the PME model for full-field light stimuli

**Figure 2:**
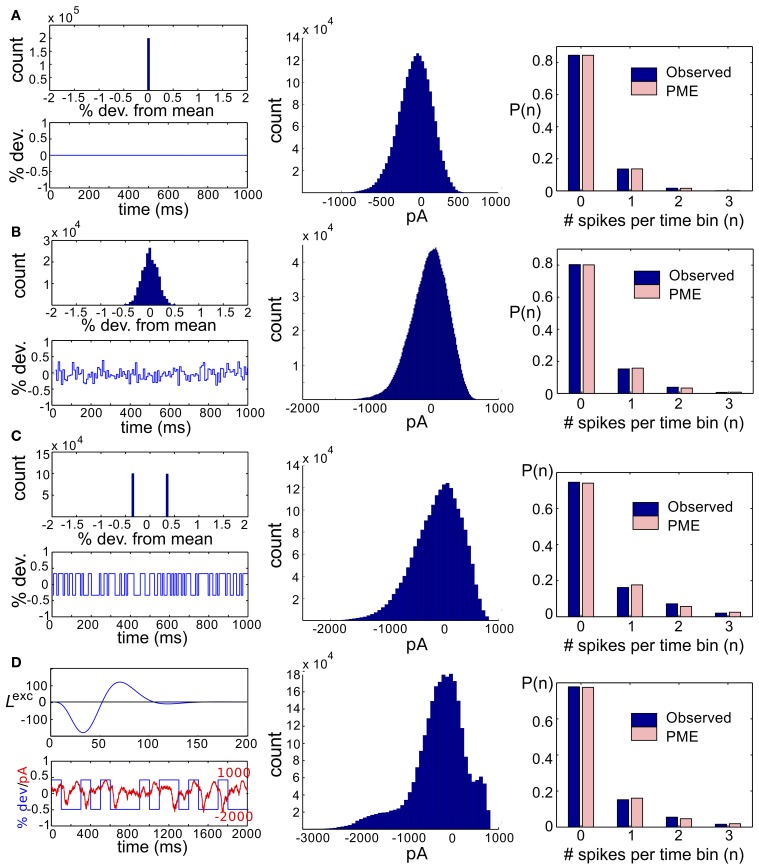
**Results for RGC simulations with constant light and full-field flicker**. **(A–C)** (Left) A histogram and time series of stimulus, (center) a histogram of excitatory conductances and (right) the resulting distribution of spiking patterns. Stimuli are shown as deviations from a baseline intensity, expressed as a fraction of the baseline. Right panels show the probability distribution on spiking patterns *P* obtained from simulation (“Observed”; dark blue), and the corresponding pairwise approximation $\tilde{P}$ (“PME”; light pink). Each row gives these results for a different stimulus condition. **(A)** No stimulus (Gaussian noise only). **(B)** Gaussian input, standard deviation 1/6, refresh rate 8 ms. **(C)** Binary input, standard deviation 1/3, refresh rate 8 ms. **(D)** Binary input, standard deviation 1/3, refresh rate 100 ms. For panel **(D)**, the data in the left panel differs. (Left, top panel) The excitatory filter *L*^exc^(*t*) (Equation 7) is shown instead of a stimulus histogram; (Left, bottom panel) the normalized excitatory conductance, as a function of time (red dashed line), is superimposed on the stimulus (blue solid). (Center) The histogram of excitatory conductances and (right) the resulting distribution of spiking patterns. Both the form of the filter and the conductance trace illustrate that the LN model that processes light input acts as a (time-shifted) high pass filter.

We start by considering networks without recurrent connectivity and with constant, full-field (i.e., spatially uniform) light stimuli. Thus, we set *s*_*k*_(*t*) = 0 for *k* = 1, 2, 3, so that the cells received only Gaussian correlated noise η^exc^_*k*_ and η^inh^_*k*_ and constant excitatory and inhibitory conductances. Time-dependent conductances were generated and used as inputs to a simulation of three model RGCs. Simulation length was sufficient to ensure significance of all reported deviations from PME fits (see section 4). We found that the spiking distributions were strikingly well-modeled by a PME fit, as shown in the righthand panel of Figure 2A; *D*_KL_(*P*, $\tilde{P}$) is 2.90 × 10^−5^ bits. This result is consistent with the very good fits found experimentally in Shlens et al. (2006) under constant light stimulation.

Next, we introduce temporal modulation into the full-field light stimuli such that each cell received the same stimulus, *s*_*k*_(*t*) = *s*(*t*), where *s*(*t*) refreshed every few milliseconds with an independently chosen value from one of several marginal distributions. For our initial set of experiments, the marginal distribution was either Gaussian (as in Ganmor et al., 2011) or binary (as used in Shlens et al., 2006). For both choices, we explored inputs with a range of standard deviations (1/16, 1/12, 1/8, 1/6, 1/4, 1/3, or 1/2 of a baseline light intensity) and refresh rates (8, 40, or 100 ms). The shared stimulus produced strong pairwise correlation between conductances of neighboring cells. However, values of *D*_KL_(*P*, $\tilde{P}$) remained small, under 10^−2^ bits in all conditions tested.

#### 2.2.3. Impact of stimulus spatial scale

**Figure 3:**
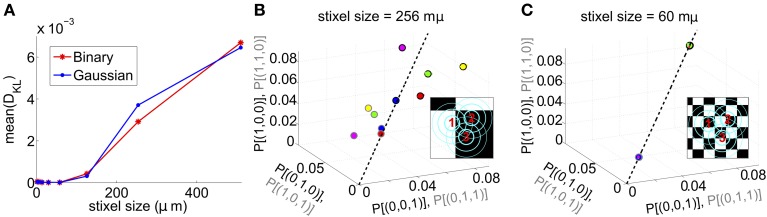
**Results for RGC simulations with light stimuli of varying spatial scale (“stixels”)**. **(A)** Average *D*_KL_(*P*, $\tilde{P}$) as a function of stixel size. Values were averaged over five stimulus positions, each with a different (random) stimulus rotation and translation; 512 μm corresponds to full-field stimuli. For the rest of the panels, data from the binary light distributions is shown; results from the Gaussian case are similar. **(B,C)** Probability of singlet and doublet spiking events, under stimulation by movies of 256 μm **(B)** and 60 μm **(C)** stixels. Event probabilities are plotted in 3-space, with the *x*, *y*, and *z* axes identifying the singlet (doublet) events 001 (011), 010 (101), and 100 (110), respectively. The black dashed line indicates perfect cell-to-cell homogeneity (e.g., *P*[(1, 0, 0)] = *P*[(0, 1, 0)] = *P*[(0, 0, 1)]). Both individual runs (dots) and averages over 20 runs (large circles) are shown, with averages outlined in black (singlet) and gray (doublet). Different colors indicate different stimulus positions. Insets: contour lines of the three receptive fields (at the 1 and 2 SD contour lines for the receptive field center; and at the zero contour line) superimposed on the stimulus checkerboard (for illustration, pictured in an alternating black/white pattern).

We next asked whether PME models capture RGC responses to stimuli with varying spatial scales. We fixed stimulus dynamics to match the two cases that yielded the highest *D*_KL_(*P*, $\tilde{P}$) under the full-field protocol: for both Gaussian and binary stimuli, we used 8 ms refresh rate and σ = 1/2. The stimulus was generated as a random checkerboard with squares of variable size; each square in the checkerboard, or *stixel*, was drawn independently from the appropriate marginal distribution and updated at the corresponding refresh rate. The conductance input to each RGC was then given by convolving the light stimulus with its receptive field, where the stimulus was positioned with a fixed rotation and translation relative to the receptive fields. This position was drawn randomly at the beginning of each simulation and held constant throughout (see insets of Figures 3B,C for examples, and section 4 for further details).

The RGC spike patterns remained very well described by PME models for the full range of spatial scales. Figure 3A shows this by plotting *D*_KL_(*P*, $\tilde{P}$) vs. stixel size. Values of *D*_KL_(*P*, $\tilde{P}$) increased with spatial scale, sharply rising beyond 128 μm, where a stixel had approximately the same size as a receptive field center, illustrating that introducing spatial scale via stixels produces even closer fits by PME models (the points at 512 μm correspond to the full-field simulations).

Values reported in Figure 3A are *averages* of *D*_KL_(*P*, $\tilde{P}$) produced by five random stimulus positions. At stixel sizes of 128 μm and 256 μm, the resulting spiking distributions differed significantly from position to position; in Figure 3B, we show the probabilities of the distinct singlet [e.g., *P*(1, 0, 0)] and doublet [e.g., *P*(1, 1, 0)] spiking events produced at 256 μm. Each stimulus position created a “cloud” of dots (identified by color); large dots show the average over 20 sub-simulations. Each sub-simulation was identified by a small dot of the same color; because the simulations were very well-resolved, most of them were contained within the large dots (and hence not visible in the figure). Heterogeneity across stimulus positioning is indicated by the distinct positioning of differently colored dots. At smaller spatial scales, the process of averaging stimuli over the receptive fields resulted in spiking distributions that were largely unchanged with stimulus position, as shown in Figure 3C, where singlet and doublet spiking probabilities are plotted for 60 μm stixels. Thus, filtered light inputs were largely homogeneous from cell to cell, as each receptive field sampled a similar number of independent, statistically identical inputs; the inset of Figure 3C shows the projection of input stixels onto cell receptive fields from an example with 60 μm stixels. The resulting excitatory conductances and spiking patterns were very close to cell-symmetric (see Figures S2B,C).

By contrast, spiking patterns showed significant heterogeneity from cell to cell when the stixel size was large, as illustrated in Figure 3B. This arises because each cell in the population may be located differently with respect to stixel boundaries, and therefore receive a distinct pattern of input activity; this is illustrated by the inset of Figure 3B, which shows the projection of input stixels onto cell receptive fields from one such simulation. However, PME models gave excellent fits to data regardless of heterogeneity in RGC responses (see Figures S2E,F); as seen in Figure 3A, over all 20 sub-simulations, and over all individual stixel positions, we found a maximal *D*_KL_(*P*, $\tilde{P}$) value of 0.00811.

#### 2.2.4. Conductance profiles and impact of stimulus filtering

Intrigued by the consistent finding of low values of *D*_KL_(*P*, $\tilde{P}$) from the RGC model circuit despite stimulation by a wide variety of highly correlated stimulus classes, we sought to further characterize the processing of light stimuli by this circuit. In particular, we examined the effects of different marginal statistics of light stimuli, standard deviation of full-field flicker, and refresh rate on the marginal distributions of excitatory conductances. We focused on excitatory conductances because they exhibit stronger correlations than inhibitory conductances in ON parasol RGCs (Trong and Rieke, 2008).

With constant light stimulation (no temporal modulation) the excitatory conductances were unimodal and broadly Gaussian (Figure 2A, middle panel). For a short refresh rate (8 ms) or small flicker size (standard deviation 1/6 or 1/4 of baseline light intensity), temporal averaging via the filter *L*^exc^ and the approximately linear form of *N*^exc^ over these light intensities produced a unimodal, modestly skewed distribution of excitatory conductances, regardless of whether the flicker was drawn from a Gaussian or binary distribution (see Figures 2B,C, center panels). For a slower refresh rate (100 ms) and large flicker size (s.d. 1/3 or 1/2 of baseline light intensity), excitatory conductances had multi-modal and skewed features, again regardless of whether the flicker was drawn from a Gaussian or binary distribution (Figure 2D). Other parameters being equal, binary light input produced more skewed conductances. While some conductance distributions had multiple local maxima, these were never well separated, with the envelope of the distribution still resembling a skewed distribution.

**Figure 4:**
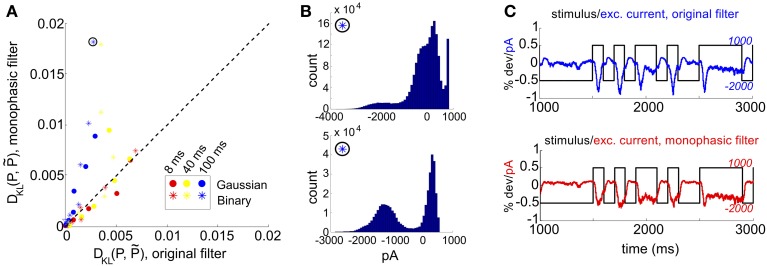
**Comparison of RGC simulations computed with the original ON parasol filter, vs. simulations using a more monophasic filter**. **(A)** *D*_KL_(*P*, $\tilde{P}$) for original vs. monophasic filter. Data is organized by stimulus refresh rate (8, 40, and 100 ms) and marginal statistics (Gaussian vs. binary). **(B)** Histograms of excitatory conductances for an illustrative stimulus class, under original (top) and monophasic (bottom) filters. The marginal statistics and refresh rate are illustrated by icons inside black circles; here, binary stimuli with refresh rate 100 ms. The input standard deviation (expressed as a fraction of baseline light intensity) was 1/2. **(C)** Time course of stimulus and resulting excitatory conductances, from simulation shown in **(B)**: original (top) vs. monophasic (bottom) filters.

The mechanism that leads to unimodal distributions of conductances, even when light stimuli are binary, is high-pass filtering—a consequence of the differentiating linear filter in Equation (7) and illustrated in Figure 2D. To demonstrate this, we constructed an alternative filter with a more monophasic shape [Equation (9), illustrated in Figure S1] and compared the excitatory conductance distributions side-by-side. We saw a striking difference in the response to long time scale, binary stimuli: the distributions produced by the monophasic filter reflected the bimodal shape of the input. Interestingly, the resulting simulation produced eight-times greater *D*_KL_(*P*, $\tilde{P}$) (Figure 4). This suggests that greater *D*_KL_(*P*, $\tilde{P}$) may occur when ganglion cell inputs are primarily characterized via monophasic filters, e.g., at low mean light levels for which the retinal circuit acts to primarily integrate, rather than differentiate over time.

In Figure 4A, we examine this effect over all full-field stimulus conditions by plotting *D*_KL_(*P*, $\tilde{P}$) from simulations with the monophasic filter, against *D*_KL_(*P*, $\tilde{P}$) from simulations in which the original filter was used with the same stimulus type. An increase in *D*_KL_(*P*, $\tilde{P}$) was observed across stimulus conditions, with a markedly larger effect for longer refresh rates. This consistent change could not be attributed to changes in lower order statistics; there was no consistent relationship between the change in pairwise model performance and either firing rate or pairwise correlations (data not shown). Instead, large effects in *D*_KL_ were accompanied by a striking increase in the bi- or multi-modality of excitatory conductances (see Figure 4B). In Figure 4C, we show an example stimulus and excitatory current trace taken from the simulation shown in Figure 4B: the monophasic filter allows the excitatory synaptic currents to track a long-timescale, bimodal stimulus with higher fidelity, transferring the bimodality of the stimulus into the synaptic currents. This finding was robust to specifics of the filtering process; we were able to reproduce the same results by designing integrating filters in different ways (data not shown).

#### 2.2.5. Recurrent connectivity in the RGC circuit

**Figure 5:**
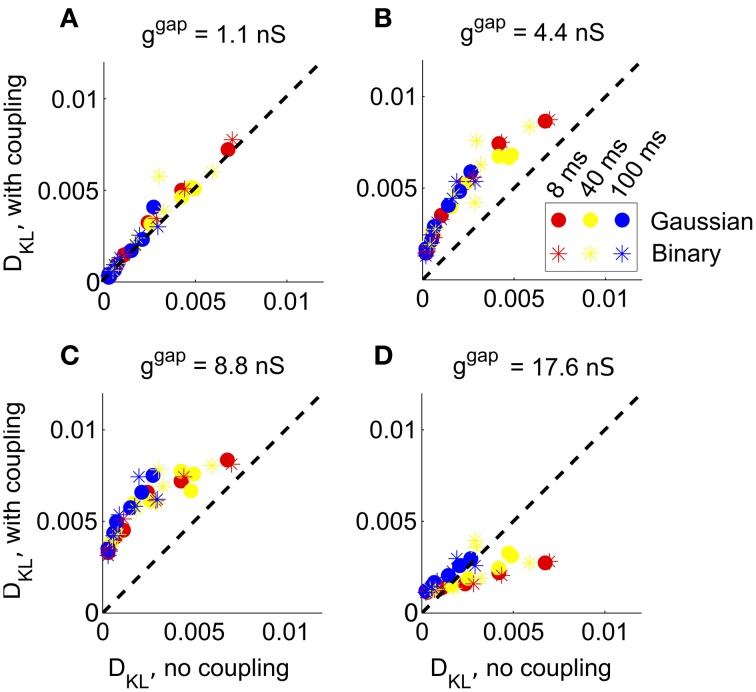
**The impact of recurrent coupling on RGC networks with full-field visual stimuli**. The strength of gap junction connections was varied from a baseline level (relative magnitude *g* = 1, or absolute magnitude *g*^gap^ = 1.1 nS) to an order of magnitude larger (*g* = 16, or *g*^gap^ = 17.6 nS). In each panel, *D*_KL_(*P*, $\tilde{P}$) obtained with coupling is plotted vs. the value obtained for the same stimulus ensemble without coupling, for each of 42 different stimulus ensembles. **(A)** *g*^gap^ = 1.1 nS (experimentally observed value); **(B)** *g*^gap^ = 4.4 nS; **(C)** *g*^gap^ = 8.8 nS; **(D)** *g*^gap^ = 17.6 nS.

We next considered the role of recurrence in shaping higher-order interactions by incorporating gap junction coupling into our simulations. We did this separately for each full-field stimulus condition described earlier. In each case, we added gap junction coupling with strengths from 1 to 16 times an experimentally measured value (Trong and Rieke, 2008), and compared the resulting *D*_KL_ with that obtained without recurrent coupling (Figure 5).

At the experimentally measured coupling strength (*g*^gap^ = 1.1 nS) itself, the fit of the pairwise model barely changed (Figure 5A) from the model without coupling. At twice the measured coupling strength (*g*^gap^ = 2.2 nS), recurrent coupling had increased higher-order interactions, as measured by larger values of *D*_KL_ for all tested stimulus conditions. Higher order interactions could be further increased, particularly for long refresh rates (100 ms), by increasing the coupling strength to four or eight times its baseline level (*g*^gap^ = 4.4 nS or *g*^gap^ = 8.8 nS; see Figures 5B,C). Consistent with the intuition that very strong coupling leads to “all-or-none” spiking patterns, *D*_KL_(*P*, $\tilde{P}$) decreased as *g*^gap^ increased further, often to a level below what was seen in the absence of coupling (Figure 5D). In summary, the impact of coupling on *D*_KL_ is maximized at intermediate values of the coupling strength. However, the impact of recurrent coupling on the maximal values of *D*_KL_ evoked by visual stimuli is small overall, and almost negligible for experimentally measured coupling strengths.

#### 2.2.6. Modeling heavy-tailed light stimuli in the RGC circuit

Finally, we repeated the full-field, recurrent, and alternate filter simulations previously described with light stimuli drawn from either Cauchy or heavy-tailed distributions: such distributions have been found to model the frequency of occurrence of luminance values in photographs of natural scenes (Ruderman and Bialek, 1994). In contrast to previous results with Gaussian and bimodal inputs, here we found very low *D*_KL_(*P*, $\tilde{P}$) over all stimulus conditions: the largest values found were more than an order of magnitude smaller than those obtained earlier. Specifically, for all conditions, we found *D*_KL_(*P*, $\tilde{P}$) < 4.5 × 10^−4^, over all 42 network realizations; for many simulations, this number did not meet a threshold for statistical significance (see section 4.1.7), indicating that *P* and $\tilde{P}$ were not statistically distinguishable. Using a more monophasic filter resulted in no apparent consistent change to *D*_KL_(*P*, $\tilde{P}$). When gap junction coupling was added, *D*_KL_(*P*, $\tilde{P}$) was maximized at an intermediate value; when *g*^gap^ = 8.8, all simulations produced a statistically significant *D*_KL_(*P*, $\tilde{P}$) ≈ 3 − 4 × 10^−3^. However, overall levels remained relatively low, roughly 1/2 the value achieved with Gaussian or binary stimuli.

**Figure 6:**
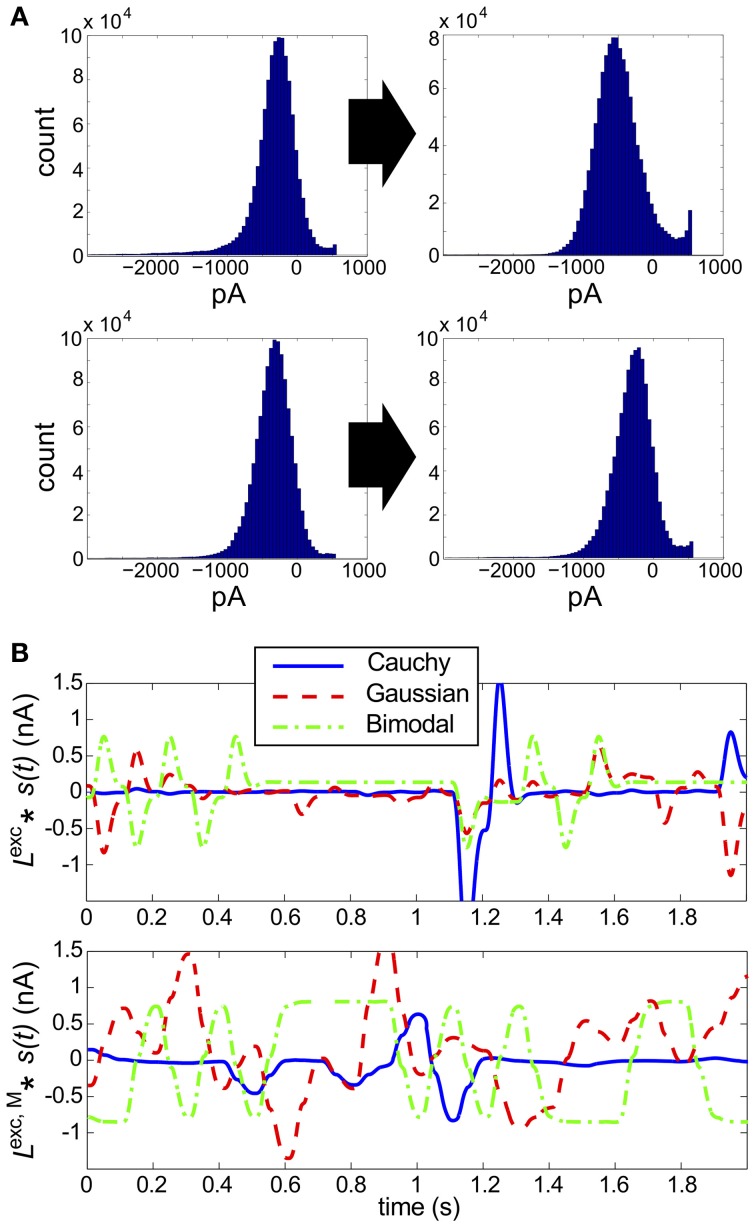
**Results for RGC simulations with heavy-tailed inputs**. **(A)** Histograms of excitatory conductances, for the original (left) vs. monophasic (right) filter. The marginal statistics are heavy-tailed skew (top) and Cauchy (bottom) inputs, and refresh rate is 40 ms for both panels. The input standard deviation (expressed as a fraction of baseline light intensity) was 1/2 for both simulations. **(B)** Sample 100 ms stimuli, filtered by the original linear filter *L*_exc_ (top) and altered, monophasic filter *L*_exc,M_(bottom). Cauchy (blue solid), Gaussian (red dashed), and bimodal (green dash-dotted) stimuli are shown.

To explain these findings, we examined the excitatory input currents: we found that over a broad range of refresh rates and stimulus variances, the marginal distributions of excitatory input conductances produced were remarkably unimodal in shape, and showed little skewness (Figure 6A). By examining the time evolution of the filtered stimuli (see Figure 6B), we see that heavy-tailed distributions allow rare, large events, but at the expense of medium-size events which explore the full range of the linear-nonlinear model used for stimulus processing (compare the blue with the red/green traces). When combined with the Gaussian background noise, this produces near-Gaussian excitatory conductances and, as may be expected from our original full-field simulations, very low *D*_KL_.

We hypothesize that the methodology of averaging over the entire stimulus ensemble may not capture the significance of rare events that may individually be detected with high fidelity: *D*_KL_ was low even for full-field, high variance stimuli, which presumably caused (infrequent) global spiking events. Additionally, an important avenue for future work would be to test the ability of our RGC model, which was trained on Gaussian stimuli, to accurately model the response of a ganglion cell to stimuli whose variance is dominated by large events. Recent work examining the adaptation of retinal filtering properties to higher-order input statistics found little evidence of adaptation; however, the stimuli used in this work incorporated significant kurtosis but not heavy tails (Tkacik et al., 2012).

#### 2.2.7. Summary of findings for RGC circuit

In summary, we probed the spiking response of a small array of RGC models to changes in light stimuli, gap junction coupling, and stimulus filtering properties, and identified two circumstances in which higher-order interactions were robustly generated in the spiking response. First, higher-order interactions were generated when excitatory currents had bimodal structure; we observed such structure when bimodal light stimuli was processed by a relatively monophasic filter. Secondly, higher-order interactions were maximized at an intermediate value of gap junction coupling; this value was, however, much larger (eight times) than the experimentally observed coupling strength.

### 2.3. A simplified circuit that explains trends in RGC cell model

#### 2.3.1. Setup and motivation

In the previous section, we developed results for a computational model tuned to a very specific cell type; we now ask whether these findings will hold for a more general class of neural circuits, or whether they are the consequence of system-specific features. To answer this question, we considered a simplified model of neural spiking: a feedforward circuit in which three spiking cells sum their inputs and spike according to whether or not they cross a threshold. Such highly idealized models of spiking have a long history in neuroscience (McCulloch and Pitts, 1943) and have been recently shown to predict the pairwise and higher-order activity of neural groups in both neural recordings and more complex dynamical spiking models (Nowotny and Huerta, 2003; Tchumatchenko et al., 2010; Yu et al., 2011; Leen and Shea-Brown, 2013).

In more detail, each cell *j* received an independent input *I*_*j*_ and a “triplet”—(global) input *I*_*c*_ that is shared among all three cells. Comparison of the total input *S*_*j*_ = *I*_*c*_ + *I*_*j*_ with a threshold Θ determined whether or not the cell spiked in that random draw. An additional parameter, *c*, identified the fraction of the total input variance σ^2^ originating from the global input; that is, *c* ≡ Var[*I*_*c*_]/Var[*I*_*c*_ + *I*_*j*_]. The global input was chosen from one of several marginal distributions, which included those used in the RGC model: Gaussian, bimodal, and heavy-tailed. The independent inputs *I*_*j*_ were, in all cases, chosen from a Gaussian distribution, consistent with our RGC model. When the common inputs are Gaussian, our model is equivalent to the Dichotomized Gaussian model previously studied by several groups (Amari et al., 2003; Macke et al., 2009, 2011; Yu et al., 2011), cf. (Tchumatchenko et al., 2010). For further details, see section 4.2.

In the RGC model large effects in *D*_KL_ were accompanied by a striking increase in the bi- or multi-modality of excitatory conductances. Why are bimodal inputs, shared across cells, able to produce spiking responses that deviate from the pairwise model? We use our simple thresholding model to provide some intuition for how bimodal common inputs to thresholding cells lead to spiking probabilities that violate the constraints (Equation 3) which must hold for the pairwise model. For example, suppose that the common input *I*_*c*_ can take on values that cluster around two separated values, μ_*A*_ < μ_*B*_, but rarely in the interval between; that is, the distribution of *I*_*c*_ is *bimodal*. If μ_*B*_ is large enough to push the cells over threshold but μ_*A*_ is not, then we see that any contribution to the right-hand side of Equation (3), *p*_2_/*p*_1_, depends only on the distribution of the independent inputs *I*_*j*_; if either one or two cells spike, then the common input must have been drawn from the cluster of values around μ_*A*_, because otherwise all three cells would have spiked.

To be concrete, let *P*[**x**] refer to the probability of spiking event **x** = (*x*_1_, *x*_2_, *x*_3_), and *P*[**x** | *I*_*c*_ ≈ μ_*A*_] refer to the probability that **x** occurs, conditioned on the event *I*_*c*_ ≈ μ_*A*_. Then

$$\begin{array}{l} P\left[\left(1,0,0\right)\right]=P\left[\left(1,0,0\right)|I_{c}\approx \mu_{A}\right]P\left[I_{c}\approx \mu_{A}\right]\\ \;+\;P[\left(1,0,0\right)|I_{c}\approx \mu_{B}]P\left[I_{c}\approx \mu_{B}\right]\\ \;=P\left[\left(1,0,0\right)|I_{c}\approx \mu_{A}\right]P\left[I_{c}\approx \mu_{A}\right] \end{array}$$

because *P*[(1, 0, 0) | I_*c*_ ≈ μ_*B*_] = 0: for the same reason,

$$P\left[\left(1,1,0\right)\right]=P\left[\left(1,1,0\right)|I_{c}\approx \mu_{A}\right]P\left[I_{c}\approx \mu_{A}\right]$$

therefore

$$\begin{array}{l} \frac{p_{2}}{p_{1}}=\frac{P\left[\left(1,1,0\right)|I_{c}\approx \mu_{A}\right]P\left[I_{c}\approx \mu_{A}\right]}{P\left[\left(1,0,0\right)|I_{c}\approx \mu_{A}\right]P\left[I_{c}\approx \mu_{A}\right]}\\ \;=\frac{P\left[\left(1,1,0\right)|I_{c}\approx \mu_{A}\right]}{P\left[\left(1,0,0\right)|I_{c}\approx \mu_{A}\right]} \end{array}$$

On the other hand,

$$\frac{p_{3}}{p_{0}}=\frac{P\left[I_{c}\approx \mu_{B}\right]+P\left[\left(1,1,1\right)|I_{c}\approx \mu_{A}\right]P\left[I_{c}\approx \mu_{A}\right]}{P\left[\left(0,0,0\right)|I_{c}\approx \mu_{A}\right]P\left[I_{c}\approx \mu_{A}\right]}.$$

By changing the relative likelihood of drawing the common input from one cluster or the other, without changing the values of μ_*A*_ and μ_*B*_ themselves (that is, change *P*[*I*_*c*_ ≈ μ_*B*_] and *P*[*I*_*c*_ ≈ μ_*A*_] but leave the conditional probabilities (e.g., *P*[(1, 0, 0) | *I*_*c*_ ≈ μ_*A*_]) fixed) one may change the ratio *p*_3_/*p*_0_ *without* changing the ratio *p*_2_/*p*_1_. Hence the constraint specifying those network responses exactly describable by PME models can be violated when the common input is bimodal.

In contrast, we may instead consider a *unimodal* common input, of which a Gaussian is a natural example. Here, the distribution of the common input *I*_*c*_ is completely described by its mean and variance; both parameters can impact the ratio *p*_3_/*p*_0_ (by altering the likelihood that the common input alone can trigger spikes) and the ratio *p*_2_/*p*_1_. Each value of *I*_*c*_ is consistent with both events *p*_1_ and *p*_2_, with the relative likelihood of each event depending on the specific value of *I*_*c*_; it is no longer clear how to separate the two events. In the following sections, we will confirm this intuition by direct evaluation of the resulting departure from pairwise statistics.

#### 2.3.2. Model input distributions

Motivated by our observations of excitatory currents that arose in the RGC model, we chose several input distributions that allow us to explore other salient features, such as symmetry and the probability of large events. A distribution is called *sub-Gaussian* if the probability of large events decays rapidly with event size, so that it can be bounded above by a scaled Gaussian distribution (see section 4). We considered two sub-Gaussian distributions; the Gaussian itself, and a skewed distribution with a sub-Gaussian tail (hereafter referred to as “skewed”). We also considered the two “heavy-tailed” distributions used as stimuli to the RGC model—the Cauchy distribution, and a skewed distribution with a Cauchy-like tail (hereafter referred to as “heavy-tailed skewed”). In these distributions, the probability of large events decays polynomially rather than exponentially.

For each choice of common input marginal, we varied the input parameters so as to explore a full range of firing rates and pairwise correlations: specifically, we varied the input correlation coefficient *c* in the range [0, 1], the *total* input standard deviation σ in the range [0, 4], and the threshold Θ in [0, 3]. In all cases the independent inputs *I*_*j*_ were chosen from a Gaussian distribution [of variance (1 − *c*) σ^2^]. For each choice of input parameters, we determine the resulting distribution on spiking states (as described in section 4) and compute the PME approximation.

#### 2.3.3. Unimodal common inputs fail to produce significant higher-order interactions in three-cell feedforward circuits

We first considered common inputs chosen from a unimodal (e.g., Gaussian) distribution. If *I*_*c*_ is Gaussian, then the joint distribution of **S** = (*S*_1_, *S*_2_, *S*_3_) is multivariate normal, and therefore characterized entirely by its means and covariances. Because the PME fit to a continuous distribution is precisely the multivariate normal that is consistent with the first and second moments, every such input distribution on **S** *exactly* coincides with its PME fit. However, even with Gaussian inputs, outputs (which are now in the binary state space {0, 1}^3^) will deviate from the PME fit (Amari et al., 2003; Macke et al., 2009). As shown below, non-Gaussian unimodal inputs can produce outputs with larger deviations. Nonetheless, these deviations are small for all cases in which inputs were chosen from a sub-Gaussian distribution, and PME models are quite accurate descriptions of circuits with a broad range of unimodal inputs.

**Figure 7:**
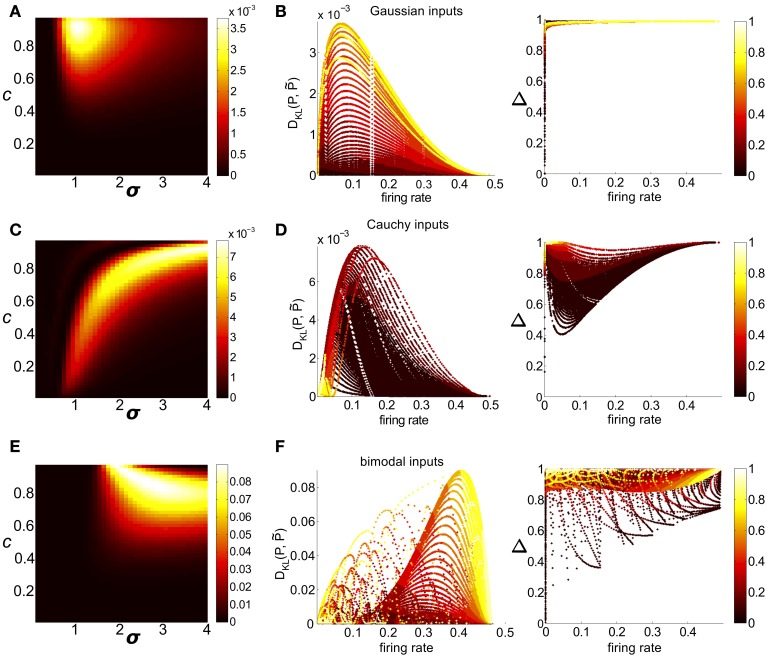
**Strength of higher-order interactions produced by the threshold model as input parameters vary, and the relationship of these higher-order interactions with other output firing statistics**. **(A)** For Gaussian common inputs: *D*_KL_(*P*, $\tilde{P}$) as a function of input correlation *c* and input standard deviation σ, for a fixed threshold Θ = 1.5. Color indicates *D*_KL_(*P*, $\tilde{P}$); see color bar for range. **(B)** For Gaussian common inputs: *D*_KL_(*P*, $\tilde{P}$) vs. firing rate (Left) and the fraction of multi-information (Δ) captured by the PME model vs. firing rate (Right). Each dot represents the value obtained from a single choice of the input parameters *c*, σ, and Θ; input parameters were varied over a broad range as described in section 2. Firing rate is defined as the probability of a spike occurring per cell per random draw of the sum-and-threshold model, as defined in Equation (16). Color indicates output correlation coefficient ρ ranging from black for ρ ∈ (0, 0.1), to white for ρ ∈ (0.9, 1), as illustrated in the color bars. **(C,D)**: as in **(A,B)**, but for Cauchy common inputs. **(E,F)**: as in **(A,B)**, but for bimodal common inputs.

We first considered circuits with either Gaussian or skewed common inputs. Over the full range of input parameters, distributions remained well fit by the pairwise model, with a maximum value of *D*_KL_(*P*, $\tilde{P}$) (of 0.0038 and 0.0035 for Gaussian and skewed inputs, respectively) achieved for high correlation values and σ comparable to threshold. In Figure 7A we illustrate these trends with a contour plot of *D*_KL_(*P*, $\tilde{P}$) for a fixed value of threshold (here, Θ = 1.5) and Gaussian common inputs (the analogous plot for skewed inputs is qualitatively very similar, Figure S3A).

Clear patterns also emerged when we viewed *D*_KL_(*P*, $\tilde{P}$) as a function of *output* spiking statistics rather than *input* statistics (as in Macke et al., 2011). Non-linear spike generation can produce substantial differences between input and output correlations; this relationship can vary widely based on the specific non-linearity (Moreno et al., 2002; de la Rocha et al., 2007; Marella and Ermentrout, 2008; Shea-Brown et al., 2008; Vilela and Lindner, 2009; Barreiro et al., 2010, 2012; Tchumatchenko et al., 2010; Hong et al., 2012). Figure 7B shows *D*_KL_(*P*, $\tilde{P}$) and Δ for all threshold values (including the data shown in Figure 7A), but now plotted with respect to the output firing rate. The data were segregated according to the Pearson's correlation coefficient ρ between the responses of cell pairs ($\rho \equiv \frac{\text{Cov}(x_{i},x_{j})}{\sqrt{\text{Var}(x_{i})\text{Var}(x_{j})}}=\frac{\hat{\rho }-\mu^{2}}{\mu (1-\mu )}$). For a fixed correlation, there was generally a one-to-one relationship between firing rate and *D*_KL_(*P*, $\tilde{P}$). For these distributions (Figure 7B, for Gaussian inputs; skewed inputs shown in Figure S3B), *D*_KL_(*P*, $\tilde{P}$) was maximized at an intermediate firing rate. Additionally, *D*_KL_(*P*, $\tilde{P}$) had a non-monotonic relationship with spike correlation: it increased from zero for low values of correlation, obtained a maximum for an intermediate value, and then decreased. These limiting behaviors agree with intuition: a spike pattern that is completely uncorrelated can be described by an independent distribution (a special case of PME model), and one that is perfectly correlated can be completely described via (perfect) pairwise interactions alone.

We next considered circuits in which inputs were drawn from one of two heavy-tailed distributions, the Cauchy distribution and a heavy-tailed skewed distribution, defined earlier. Here, distinctly different patterns emerge: for a fixed Θ, *D*_KL_(*P*, $\tilde{P}$) is maximized in regions of high input correlation and high input variance σ, but relatively high values of *D*_KL_ are achievable across a wide range of input values (see Figure 7C for Cauchy inputs; heavy-tailed skewed in Figure S3C). However, the maximum achievable values of *D*_KL_ were achieved at intermediate *output* correlations ρ ≈ 0.4 (see Figure 7D for Cauchy inputs; heavy-tailed skewed shown in Figure S3D); this suggests that high input correlations do not result in high output correlations.

This somewhat unintuitive finding may be explained by the structure of the PDF of a heavy-tailed common input, which favors (infrequent) large events at the expense of medium-size events. For instance, the probability that a Cauchy input is above a given threshold (*P*[*I*_*c*_ > Θ > **E**[*I*_*c*_]]) is often much smaller than for a Gaussian distribution of the same variance. However, an input can trigger at best one single spiking event regardless of size: therefore a Cauchy common input generates fewer correlated spiking events with larger inputs, while a Gaussian common input triggers correlated spiking events with smaller, but more frequent, input values. As a result, heavy-tailed inputs are unable to explore the full range of output firing statistics: Figure 7D shows that high output correlations only occur at very low firing rates. Overall, *D*_KL_(*P*, $\tilde{P}$) reaches higher numerical values than for sub-Gaussian inputs, possibly reflecting the higher-order statistics in the input. However, the maximal *D*_KL_(*P*, $\tilde{P}$) attained still falls far short of exploring the full range of possible values (compare with Figure 1B).

Finally, we examine the behavior of the *strain*, which quantifies both the magnitude and sign of deviation from the pairwise model (see Ohiorhenuan and Victor, 2010). It has been previously observed that the strain is negative for the DG model (Macke et al., 2011), a condition that has been related to sparsity of the neural code and with which our results agree (data not shown). However, we found that any other choice of input marginal statistics, both positive and negative values are seen; for heavy-tailed common inputs, positive values predominated except at very low firing rates.

#### 2.3.4. Bimodal triplet inputs can generate higher-order interactions in three-cell feedforward circuits

Having shown that a wide range of unimodal common inputs produced spike patterns that are well-approximated by PME fits, we next examined bimodal common inputs. Such inputs substantially increased departures from PME fits in the ganglion cell models described above. As in the previous section, we varied *c*, σ, and Θ so as to explore a full range of firing rates and pairwise correlations.

As a function of input parameter values, *D*_KL_(*P*, $\tilde{P}$) is maximized for large input correlation and moderate input variance σ^2^ [see Figure 7E, which illustrates *D*_KL_(*P*, $\tilde{P}$) for a fixed threshold Θ = 1.5]. Figure 7F shows *D*_KL_(*P*, $\tilde{P}$) values as a function of the firing rate and pairwise correlation elicited by the full range of possible bimodal inputs. We see that *D*_KL_(*P*, $\tilde{P}$) is maximized at an intermediate (but relatively high: ν ≈ 0.4) firing rate, and for intermediate-to-large correlation values (ρ ≈ 0.6 − 0.8).

We find distinctly different results when we view Δ (Equation 1), for these same simulations, as a function of output spiking statistics (right panels of Figures 7B,D,F). For unimodal, sub-Gaussian distributions (Figure 7B), Δ is very close to 1, with the few exceptions at extreme firing rates. For heavy-tailed and bimodal inputs (Figures 7D,F), Δ may be appreciably far from 1 (as small as 0.5) with the smallest numbers (suggesting a poor fit of the pairwise model) occurring for low correlation ρ. This highlights one interesting example where these two metrics for judging the quality of the pairwise model, *D*_KL_(*P*, $\tilde{P}$) and Δ, yield contrasting results.

Finally, we emphasize that while bimodal inputs can produce greater higher-order interactions than unimodal inputs, the values of *D*_KL_(*P*, $\tilde{P}$) accessible by feedforward circuits with global inputs remain far below their upper bounds at any given firing rate. The maximal values of *D*_KL_(*P*, $\tilde{P}$) reached by Cauchy and heavy-tailed skewed inputs were 0.0078 and 0.0153; bimodal common inputs reached a maximal value of 0.091. This is an order of magnitude smaller than possible departures among symmetric spike patterns (compare Figure 1B). The difference is illustrated in Figure S4, which compares the *D*_KL_(*P*, $\tilde{P}$) values obtained in the thresholding model and those obtained by direct exhaustive search at each firing rate by superposing the datapoints on a single axis.

#### 2.3.5. Mathematical analysis of unimodal vs. bimodal effects

The central finding above is that circuits with bimodal inputs can generate significantly greater higher-order interactions than circuits with unimodal inputs. To probe this further, we investigated the behavior of *D*_KL_(*P*, $\tilde{P}$) for the feedforward threshold model with a perturbation expansion in the limit of small common input. We found that as the strength of common input signals increased, circuits with bimodal inputs diverged from the PME fit more rapidly than circuits with unimodal inputs; the full calculation is given in the Appendix. In brief, we determined the leading order behavior of *D*_KL_(*P*, $\tilde{P}$) in the strength *c* of (weak) common input. *D*_KL_(*P*, $\tilde{P}$) depended on *c*^3^ for unimodal distributions, i.e., the low order terms in *c* dropped out; for symmetric unimodal distributions, such as a Gaussian, *D*_KL_(*P*, $\tilde{P}$) grew as *c*^4^. For bimodal distributions, *D*_KL_(*P*, $\tilde{P}$) grew as *c*^2^. Because of the *c*^2^ dependence, rather than *c*^3^ or *c*^4^, as the strength of common input signals *c* increases, circuits with bimodal inputs are predicted to produce greater deviations from their PME fits.

#### 2.3.6. Impact of recurrent coupling

We next modified our thresholding model to incorporate the effects of recurrent coupling among the spiking cells. To mimic gap junction coupling in the RGC circuit, we considered all-to-all, excitatory coupling, and assumed that this coupling occurs on a faster timescale compared with the timescale over which inputs arrive at the cells.

Our previous model was extended as follows: if the inputs arriving at each cell elicited any spikes, there was a second stage at which the input to each neuron receiving a connection from a spiking cell was increased by an amount *g*. This represented a rapid depolarizing current, assumed for simplicity to add linearly to the input currents. If the second stage resulted in additional spikes, the process was repeated: recipient cells received an additional current *g*, and their summed inputs were again thresholded. The sequence terminated when no new spikes occurred on a given stage; e.g., for *N* = 3, there were a maximum of three stages. The spike pattern recorded on a given trial was the total number of spikes generated across all stages.

**Figure 8:**
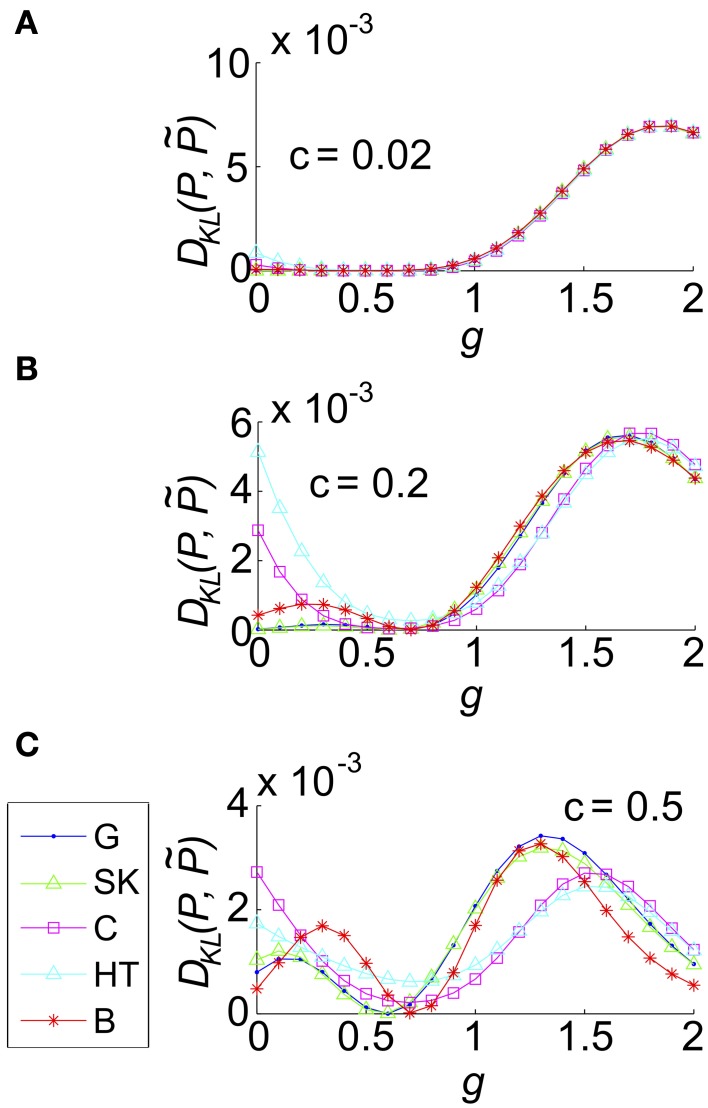
**The impact of recurrent coupling on the three-cell sum-and-threshold model**. Each plot shows *D*_KL_(*P*, $\tilde{P}$) as a function of *g*, for a specific value of the correlation coefficient. In all panels, input standard deviation σ = 1, threshold Θ = 1.5, *N* = 3 and symbols are as described in the legend for **(C)**. Abbreviations in the legend denote the marginal distribution of the common input: G, Gaussian; SK, skewed; C, Cauchy; HT, heavy-tailed skewed; B, bimodal. **(A)** For input correlation *c* = 0.02, **(B)** *c* = 0.2, and **(C)** *c* = 0.5.

We then explored the impact of varying *g* for a single representative value of σ and Θ, and several values of the correlation coefficient *c*. We found that as *g* increased *D*_KL_(*P*, $\tilde{P}$) varied smoothly, reflecting the underlying changes in the spike count distribution. For small *c* (*c* = 0.02 shown in Figure 8A), where the variance of common input is very small, the results varied little by input type: for all input types *D*_KL_(*P*, $\tilde{P}$) reached an interior maximum near *g* ≈ 1.7. As *c* increases, the distinctions between inputs types become apparent (Figures 8B,C show *c* = 0.2, 0.5, respectively): for most input types and values of *c*, the value of *D*_KL_(*P*, $\tilde{P}$) reaches an interior maximum that exceeds its value without coupling (i.e., *g* = 0). However, overall values of *D*_KL_(*P*, $\tilde{P}$) remained modest, never exceeding 0.01 across the values explored here.

#### 2.3.7. Summary of findings for simplified circuit model

We examined a highly idealized model of neural spiking, so as to explore the generality of our earlier findings in a small array of RGC models. We found that our main results from the RGC model—that higher-order interactions were most significant when inputs had bimodal structure, and that when fast excitatory recurrence was added to the circuit, higher-order interactions were maximized at an intermediate value of the recurrence strength—persisted in this simplified model. Moreover, we were able to show that the first of these findings is general, in that it holds over a complete exploration of parameter space.

### 2.4. Scaling of higher-order interactions with population size

The results above suggest that unimodal, rather than bimodal, input statistics contribute to the success of PME models. Next, we examined whether this conclusion continues to hold when we increase network size. The permutation-symmetric architectures we have considered so far can be scaled up to more than three cells in several natural ways; for example, we can study *N* cells with a global common input.

**Figure 9:**
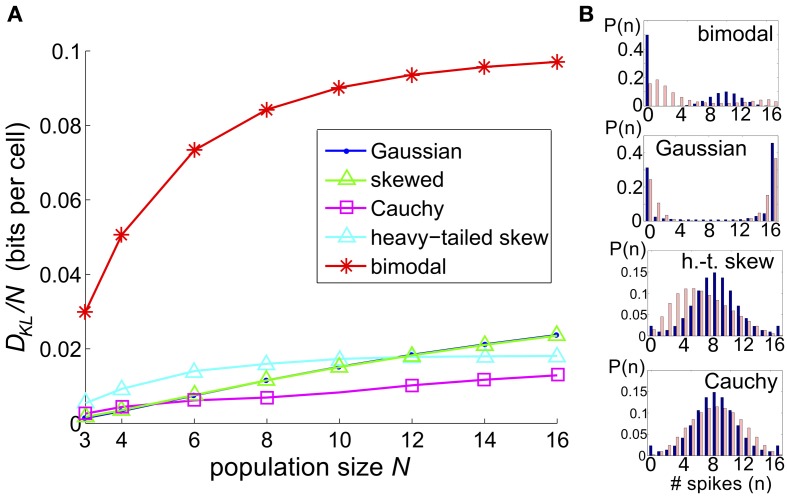
**The significance of higher-order interactions increases with network size**. **(A)** Normalized maximal deviation, *D*_KL_(*P*, $\tilde{P}$)/*N*, from the PME fit for the thresholding circuit model as network size *N* increases. For each *N* and common input distribution type, possible input parameters were in the following ranges: input correlation *c* ∈ [0, 1], input standard deciation σ ∈ [0, 4], and threshold Θ ∈ [0, 3]. **(B)** Example sample distributions for different types of common input: from top, bimodal, Gaussian, heavy-tailed skew, and Cauchy common inputs. For each input type, the distribution that maximized *D*_KL_(*P*, $\tilde{P}$) for *N* = 16 is shown. Each distribution is illustrated with a bar plot contrasting the probabilities of spiking events in the true (dark blue) vs. pairwise maximum entropy (light pink) distributions.

We considered a sequence of models in which a set of *N* threshold spiking units received global input *I*_*c*_ [with mean 0 and variance σ^2^*c*] and an independent input *I*_*j*_ [with mean 0 and variance σ^2^ (1 − *c*)]. As for the three-cell network models considered previously, the output of each cell was determined by summing and thresholding these inputs. Upon computing the probability distribution of network outputs (section 4), we fit a PME distribution. Again, we explored a range of σ, *c*, and Θ and recorded the maximum value of *D*_KL_(*P*, $\tilde{P}$) between the observed distribution *P* and its PME fit $\tilde{P}$. Figure 9 shows this *D*_KL_/*N* [i.e., entropy per cell (Macke et al., 2009)] for each class of marginal distributions.

We found that the maximum *D*_KL_(*P*, $\tilde{P}$)/*N* increased roughly linearly with *N* for Gaussian, skewed and Cauchy inputs; for heavy-tailed skew and bimodal inputs, *D*_KL_(*P*, $\tilde{P}$)/*N* appeared to saturate after an initial increase (Figure 9). The relative ordering for unimodal inputs shifted as *N* increased; as *N* → 16, the maximal achievable *D*_KL_(*P*, $\tilde{P}$) for sub-Gaussian inputs overtook the values for heavy-tailed inputs. At all values of *N*, the values for Gaussian and skewed inputs tracked one another closely. Regardless, the values for all unimodal inputs remained substantially below the maximal value achievable for bimodal inputs. Figure 9B shows that the probability distributions produced by these inputs qualitatively agree with this trend: departures from PME were more visually pronounced for global bimodal inputs than for global unimodal inputs. In addition, the distributions for heavy-tailed and sub-Gaussian inputs differed qualitatively, offering a potential mechanism for different scaling behavior. Using the relationship between *D*_KL_ and likelihood ratios (described in section 2.1), at *N* = 16, the value *D*_KL_/*N* ≈ 0.1 for bimodal global inputs corresponds to a likelihood ratio of 0.33 that a single draw from *P* (single network output) in fact came from the PME fit $\tilde{P}$ rather than from *P*; a likelihood <0.01 is reached for four draws.

**Figure 10:**
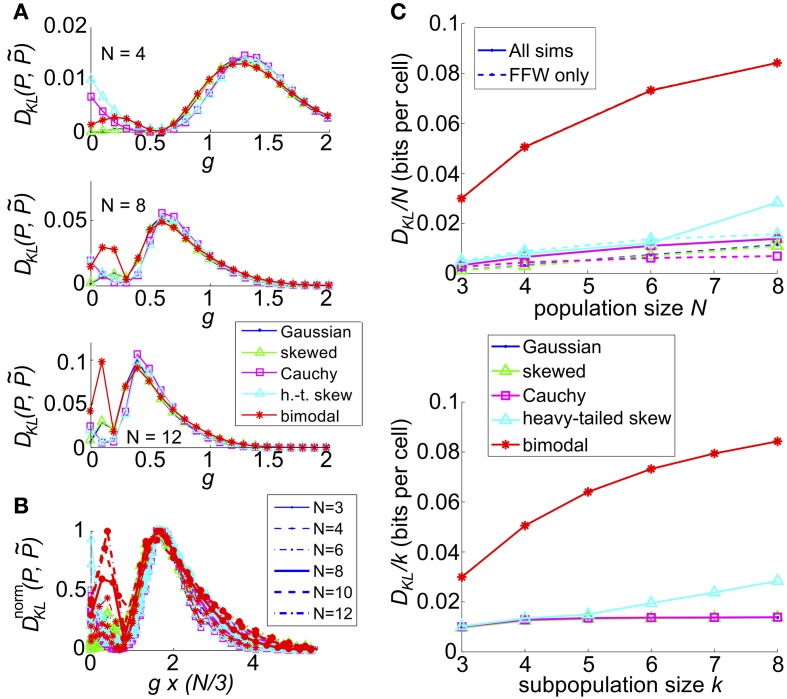
**The impact of recurrent coupling on the sum-and-threshold model, for increasing population size**. **(A)** *D*_KL_(*P*, $\tilde{P}$) as a function of the coupling coefficient, *g*, for a specific value of population size *N*. In all plots, input standard deviation σ = 1, threshold Θ = 1.5 and input correlation *c* = 0.2. From top: *N* = 4; *N* = 8; *N* = 12. **(B)** *D*^norm^_KL_(*P*, $\tilde{P}$) as a function of the coupling coefficient, *g*, for populations sizes *N* = 3-12. For each curve, *D*_KL_(*P*, $\tilde{P}$) was scaled by its maximal value and plotted as a function of the scaled coupling coefficient, g^*^N/3, to illustrate a universal scaling with effective coupling strength. The line style of each curve indicates the population size *N*, as listed in the legend. The marker and line color indicate the common input marginal, as listed in the legend for **(A)**. **(C)** (Top) Maximal value of *D*_KL_(*P*, $\tilde{P}$)/*N*, achieved over a survey of parameter values *c*, σ, Θ, and *g*, as a function of the population size *N* (solid lines). For each input marginal type, a second curve shows the maximal value obtained over only feed-forward simulations (*g* = 0; dashed lines). The marker and line color indicate the common input marginal, as listed in the legend for **(A)**. (Bottom) Maximal value of *D*_KL_(*P*, $\tilde{P}$)/*k*, achieved over a survey of parameter values *c*, σ, Θ, and *g*, as a function of the *subsample* population size *k*. Data was subsampled from the *N* = 8 data shown in the top panel, by restricting analysis to *k* out of *N* cells.

We next extended our model with recurrent coupling to *N* > 3 cells. In addition to the parameters for the uncoupled network, we varied the coupling strength, *g*, for each type of input. As in the *N* = 3 network, coupling was all-to-all. As for the small networks explored in an earlier section, *D*_KL_(*P*, $\tilde{P}$) generally peaked at an intermediate value of the coupling strength *g*; however, the value of *g* decreased as population size *N* increased (illustrated in Figure 10A, for *c* = 0.2). This may be attributed to the increased potential impact of recurrence at larger population sizes; as *N* increases, the number of potential *additional* spikes that may be triggered increases; consequently the average recurrent excitation received by each cell increases, and therefore the probability that one or two spikes will trigger a cascade to *N* spikes. In Figure 10B we demonstrate that the impact of this effect may be captured by plotting *D*_KL_(*P*, $\tilde{P}$) as a function of an *effective* coupling parameter, g^*^N/3. Here, we plot the curves for six population sizes (*N* = 3, 4, 6, 8, 10, and 12) and five common input types; each curve was scaled by normalizing *D*_KL_(*P*, $\tilde{P}$) by its maximum value. For many sets of parameter values, the resulting curves line up remarkably well, suggesting a universal scaling with the effective coupling parameter.

We also explored the overall possible impact of recurrence on higher-order interactions, by surveying a range of circuit parameters *c*, σ, Θ and *g*. The top panel of Figure 10C shows the maximal *D*_KL_(*P*, $\tilde{P}$) per neuron, for each type of input, up to population size *N* = 8. For unimodal inputs, recurrent coupling increased the available range of higher-order interactions modestly, compared with the range achieved with purely feedforward connections; however, these values remained significantly lower than those achieved for bimodal inputs.

Finally, we considered how higher-order interactions scale with population sampling size. The spike pattern distributions used to generate the last column of data points (*N* = 8) in the top panel of Figure 10C were reanalyzed by sub-sampling the spike pattern distributions on *k* < 8 cells. In each case, we chose our sub-population to be *k* nearest neighbors (for our setup, any subset of *k* cells is statistically identical). In the bottom panel of Figure 10C, we show the maximal value of *D*_KL_(*P*, $\tilde{P}$) per sub-sampled cell achieved over all input parameters (the curves for Gaussian, skewed and Cauchy inputs are so close together so as to be visually indistinguishable). This number increases or remains steady as *k* increases, indicating that sub-sampling a coupled network will depress the apparent higher-order interactions in the output spiking pattern.

To summarize, the greater impact of bimodal vs. unimodal input statistics on maximal values of *D*_KL_(*P*, $\tilde{P}$) persists in circuits with *N* = 3 cells up to *N* = 16 cells. Overall, for the circuit parameters producing maximal deviations from PME fits, it becomes easier to statistically distinguish between spiking distributions and their PME fits as the number of cells increases in feedforward networks.

## 3. Discussion

We used mechanistic models to identify input patterns and circuit mechanisms which produce spike patterns with significant higher-order interactions—that is, with substantial deviations from predictions under a PME model. We focused on a tractable setting of small, symmetric circuits with common inputs. This revealed several general principles. First, we found that these circuits produced outputs that were much closer to PME predictions than required for a general spiking pattern. Second, bimodal input distributions produced stronger higher-order interactions than unimodal distributions. Third, recurrent excitatory or gap junction coupling could produce a further, moderate increase of higher-order correlations; the effect was greatest for coupling of intermediate strength.

These general results held for both an abstract threshold-and-spike model and for networks of non-linear integrate-and-fire units based on measured properties of one class of RGCs. Together with the facts that ON parasol cell filtering suppresses bimodality in light input, and that coupling among ON parasol cells is relatively weak, our findings provide an explanation for why their population activity is well captured by PME models.

### 3.1. Comparison with empirical studies

How do our maximum entropy fits compare with empirical studies? In terms of *D*_KL_(*P*, $\tilde{P}$)—equivalently, the logarithm of the average relative likelihood that a sequence of data drawn from *P* was instead drawn from the model $\tilde{P}$—numbers obtained from our RGC models are very similar to those obtained by *in vitro* experiments on primate RGCs (Shlens et al., 2006, 2009). For example, in a survey of 20 numerical experiments under constant light conditions (each of length 100 ms, with spikes binned in 10 ms intervals), we find that *D*_KL_(*P*, $\tilde{P}$) ranges between 0 and 0.00029: similarly excellent fits were found by Shlens et al. (2006) (in which cell triplets were stimulated by constant light for 60 s with spikes binned at 10 ms), with one example given of 0.0008 (inferred from a reported likelihood ratio of 0.99944). These values can increase by an order of magnitude under full-field stimulation, as well as spatio-temporally varying stixel simulations (bounded above by 0.007). We can view the 60 μm stixel simulations as a model of the checkerboard experiments of Shlens et al. (2006), for which close fits by the PME distribution were also observed (likelihood numbers were not reported). Similarly, the values of Δ produced by our RGC model are close to those found by Schneidman et al. (2006); Shlens et al. (2006) under comparable stimulus conditions. We obtain Δ = 99.5% (for cell group size *N* = 3) under constant illumination, which is near the range reported by Shlens et al. (2006) for the same bin size and stimulus conditions (98.6 ± 0.5, *N* = 3 − 7). For full-field stimuli we find a range of numbers from 95.7% to 99.3% (*N* = 3).

With regard to the circuit mechanisms behind these excellent fits by pairwise models, the findings that most directly address the experimental settings of Shlens et al. (2006, 2009), are (1) the finding that in the threshold model, unimodal inputs generate minimal higher-order interactions, compared to bimodal inputs, and (2) the particular stimulus filtering properties of parasol cells can suppress bimodality that may be present in an input stimulus, resulting in a unimodal distribution of input currents. First, we believe that unimodal inputs are consistent with the white-noise checkboard stimuli used in Shlens et al. (2006, 2009), where binary pixels were chosen to be small relative to the receptive field size; averaged over the spatial receptive field, they would be expected to yield a single Gaussian input by the central limit theorem. Second, temporal filtering may contribute to receipt of unimodal conductance inputs by cells for the full-field binary flicker stimuli that are delivered in Schneidman et al. (2006). With the 16.7 ms refresh rate used there, under the assumption that the filter time-scale of the cells studied in that paper is roughly similar to that of the ON parasol cell we consider, the filter would average a binary (and hence bimodal) stimulus into a unimodal shape (see Figure 2C, for example).

The simple threshold models that we have considered, meanwhile, give us a roadmap for how circuits could be driven in such a way as to lower Δ. The right columns of Figures 7B,D,F show Δ plotted as a function of firing rate for circuits of *N* = 3 cells receiving global common inputs; we observe that Δ ≈ 1 for Gaussian inputs over a broad range of firing rates and pairwise correlation coefficients, but that values of Δ can be depressed by 25–50% in the presence of a bimodal common input. Indeed, Shlens et al. (2006) showed that adding global bimodal inputs to a purely pairwise model can lead to a comparable departure in Δ. Our results are consistent with this finding, and explicitly demonstrate that the bimodality of the inputs—as well as their global projection—are characteristics that lead to this departure.

### 3.2. Consequences for specific neural circuits

Our results make predictions about when neural circuits are likely to generate higher-order interactions. A comprehensive study of our simple thresholding model shows that bimodal inputs generate greater beyond-pairwise interactions than unimodal inputs. This result can be extended to other circuits where a clear input–output relationship exists, and be used to predict higher-order correlations by analyzing the impact of stimulus filtering on a statistically defined class of inputs. For example, the effect holds in our model of primate ON parasol cells, where a biphasic filter suppresses bimodality in a stimulus with a timescale matched to that of the filter. We can use these results to extrapolate to other classes of RGCs or other stimulus conditions in which filters are less biphasic (Victor, 1999). Indeed, when we process long time-scale bimodal inputs through a preliminary model of the midget cell circuit, stimulus bimodality is no longer suppressed and is associated with higher-order interactions (see Figure 4). We predict that greater higher-order interactions will be found for stimuli or RGC circuits that elicit bimodal activity that is thresholded when generating spikes—in comparison to the parasol circuits and stimuli studied in Shlens et al. (2006, 2009). We believe that this principle will be further applicable in other sensory systems.

We found that recurrent excitatory connections further increase higher-order interactions, which are maximized at an intermediate recurrence strength; in particular, when the strength of an excitatory recurrent input was comparable to the distance between rest and threshold (Figure 8). For the primate ON parasol cells we considered, the experimentally measured strength of gap junction coupling would lead to an estimated membrane voltage jump of ≈ 1 mV in response to the firing of a neighboring RGC, while the voltage distance between the resting voltage and an approximate threshold is about 5–10 mV (Trong and Rieke, 2008). Consistent with this estimate, we found that in our ON parasol cell model, higher-order interactions were maximized when the strength of excitatory recurrence was eight times its experimentally measured value. The experimentally measured values of recurrence had little or no effect on higher-order interactions. We anticipate that this result may be used to predict whether recurrent coupling plays a role in generating higher-order interactions in other circuits where the average voltage jump produced by an electrical or synaptic connection can be measured.

To apply our findings to real circuits, we must also consider population size. A measurement from a neural circuit, in most cases, will be a subsample of a much larger, complete circuit. We addressed this question where it was computationally more tractable, for the thresholding model. Here, we found that the impact of higher-order interactions, as measured by entropy per cell unaccounted for by the pairwise model (*D*_KL_/*k*), increases moderately as subsample size *k* increases. Since recurrent connectivity in our model is truly global, this is consistent with the suggestion of Roudi et al. (2009a) and others that the entropy can be expected to scale extensively with population size *N*, once *N* significantly exceeds the true spatial connectivity footprint: we may see different results with limited, local connectivity.

### 3.3. Scope and open questions

There are many aspects of circuits left unexplored by our study. Prominent among these is heterogeneity. Only a few of our simulations produce heterogeneous inputs to model RGCs, and all of our studies apply to cells with identical response properties. This is in contrast to studies such as Schneidman et al. (2006), which examine correlation structures among multiple cell types. For larger networks, feedforward connections with variable spatial profiles also occur, between the extremes of independent and global input connections examined here. It is also possible that more complex input statistics could lead to greater higher-order interactions (Bethge and Berens, 2008). Finally, Figure 9 indicates that some trends in *D*_KL_(*P*, $\tilde{P}$) vs. N appear to become non-linear for *N* ⪆ 10; for larger networks, our qualitative findings could change.

Our study also leaves largely open the role of different retinal filters in generating higher-order interactions. We have found that the specific filtering properties of ON parasol cells suppress bimodality in light inputs, suggesting that other classes of RGCs, such as midget cells, may produce more robust higher-order interactions (compare panels in Figure 4B). This predicts a specific mechanism for the development of higher-order interactions in preparations that include multiple classes of ganglion cells (Schneidman et al., 2006). For a complete picture, future studies will also need to account for the possible adaptation of stimulus filters in response to higher-order stimulus characteristics (Tkacik et al., 2012); we did not consider the latter effect here, where our filter was fit to the response of a cell to Gaussian stimuli with specific mean and variance. An allied possibility is that multiple filters will be required, as was found when fitting the responses of salamander retinal cells to LN models (Fairhall et al., 2006). Distinguishing the roles of linear filters vs. static non-linearities in determining which stimulus classes will give the greatest higher-order correlations is another important step. Finally, we considered circuits with a single step of inputs and simple excitatory or gap junction coupling; a plethora of other network features could also lead to higher-order interactions, including multi-layer feedforward structures, together with lateral and feedback coupling. We speculate that, in particular, such mechanisms could contribute to the higher-order interactions found in cortex (Tang et al., 2008; Montani et al., 2009; Ohiorhenuan et al., 2010; Oizumi et al., 2010; Koster et al., 2013).

A final outstanding area of research is to link tractable network mechanisms for higher-order interactions with their impact (or lack of impact) on information encoded in neural populations (Kuhn et al., 2003; Montani et al., 2009; Oizumi et al., 2010; Ganmor et al., 2011; Cain and Shea-Brown, 2013). A simple starting point is to consider rate-based population codes in which each stimulus produces a different “tuned” average spike count (see for e.g., chapter 3 of Dayan and Abbot, 2001). One can then ask whether spike responses can be more easily decoded to estimate stimuli for the full population response (i.e., *P*) to each stimulus or for its pairwise approximation ($\tilde{P}$). In our preliminary tests where higher-order correlations were created by inputs with bimodal distributions, we found examples where decoding of *P* vs. $\tilde{P}$ differed substantially. However, a more complete study would be required before general conclusions about trends and magnitudes of the effect could be made; such a study would include complementary approach in which the full spike responses *P* are themselves decoded via a “mismatched” decoder based on the pairwise model $\tilde{P}$ (Oizumi et al., 2010). Overall, we hope that the present paper, as one of the first that connects circuit mechanisms to higher-order statistics of spike patterns, will contribute to future research that takes these next steps.

## 4. Materials and methods

### 4.1. Experimentally-based model of a RGC circuit

We model the response of a individual RGC using data collected from a representative primate ON parasol cell, following methods in Murphy and Rieke (2006); Trong and Rieke (2008). Similar response properties were observed in recordings from 16 other cells. To measure the relationship between light stimuli and synaptic conductances, the retina was exposed to a full-field, white noise stimulus. The cell was voltage clamped at the excitatory (or inhibitory) reversal potential *V*_*E*_ = 0 mV (*V*_*I*_ = −60 mV), and the inhibitory (or excitatory) currents were measured in response to the stimulus. These currents were then turned into equivalent conductances by dividing by the driving force of ± 60 mV; in other words

$$\begin{array}{l} g^{\text{exc}}=I^{\text{exc}}/(V-V_{E});\;V-V_{E}=-60\text{ mV}\\ g^{\text{inh}}=I^{\text{inh}}/(V-V_{I});\;V-V_{I}=60\text{ mV} \end{array}$$

The time-dependent conductances *g*^exc^ and *g*^inh^ were now injected into a different cell using a dynamic clamp procedure (i.e., the input current was varied rapidly to maintain the correct relationship between the conductance and the membrane voltage) and the voltage was measured at a resolution of 0.1 ms.

#### 4.1.1. Stimulus filtering

To model the relationship between the light stimulus and synaptic conductances, the current measurements *I*^exc^ and *I*^inh^ were fit to a linear-nonlinear model:

$$\begin{array}{l} g^{\text{exc}}(t)=N^{\text{exc}}\left[L^{\text{exc}}*s(t)+\eta^{\text{exc}}\right],\\ g^{\text{inh}}(t)=N^{\text{inh}}\left[L^{\text{inh}}*s(t)+\eta^{\text{inh}}\right] \end{array}$$

where *s* is the stimulus, *L*^exc^ (*L*^inh^) is a linear filter, *N*^exc^ (*N*^inh^) is a non-linear function, and η^exc^ (η^inh^) is a noise term. The linear filter was fit by the function

(7) $$L^{\text{exc}}(t)=P_{\text{exc}}{\left(t/\tau_{\text{exc}}\right)}^{n_{\text{exc}}}\exp\left(-t/\tau_{\text{exc}}\right)\sin\left(2\pi t/T_{\text{exc}}\right)$$

and the non-linear filter by the polynomial

(8) $$N^{\text{exc}}(x)=A_{\text{exc}}x^{2}+B_{\text{exc}}x+C_{\text{exc}}.$$

Fits minimized the mean-square distance between model and data. *L*^inh^ and *N*^inh^ were fit using the same parametrization.

The noise terms η^exc^_*k*_, η^inh^_*k*_ were fit to reproduce the statistical characteristics of the residuals from this fitting. We simulated the noise terms η^exc^ and η^inh^ using Ornstein–Uhlenbeck processes with the appropriate parameters; these were entirely characterized by the mean, standard deviation, and time constant of autocorrelation τ_η,exc_ (τ_η, inh_), as well as pairwise correlation coefficients for noise terms entering neighboring cells. The noise correlation coefficients were estimated from the dual recordings of Trong and Rieke (2008).

Linear filter parameters computed (also listed in Table 1) were *P*_exc_ = −8 × 10^4^ s^−1^, *n*_exc_ = 3.6, τ_exc_ = 12 ms, *T*_exc_ = 105 ms, and *P*_inh_ = −1.8 × 10^5^ s^−1^, *n*_inh_ = 3.0, τ_inh_ = 16 ms, *T*_inh_ = 120 ms. Non-linearity parameters were *A*_exc_ = −8.3 × 10^−7^ nS, *B*_exc_ = 7 × 10^−3^ nS, *C*_exc_ = −0.95 nS, and *A*_inh_ = 1.67 × 10^−6^ nS, *B*_inh_ = 6.2 × 10^−3^ nS, *C*_inh_ = 4.17 nS. Noise parameters were measured to be mean(η^exc^_*k*_) = 30, std(η^exc^_*k*_) = 500, τ_η,exc_ = 22 ms, and mean(η^inh^_*k*_) = −1200, std (η^inh^_*k*_) = 780, τ_η,inh_ = 33 ms. In addition, excitatory (inhibitory) noise to different cells η^exc^_*k*_, η^exc^_*j*_ (η^inh^_*k*_, η^inh^_*j*_) had a correlation coefficient of 0.3 (0.15).

**Table 1:** **Parameters used to model the transformation of stimuli into synaptic conductances for the RGC model, as described in Equations (7–9)**.

**Model (MOD)**	***P*_MOD_ (s^−1^)**	**τ_MOD_ (ms)**	***n*_MOD_**	***T*_MOD_ (ms)**	***A*_MOD_ (nS)**	***B*_MOD_ (nS)**	***C*_MOD_ (nS)**
exc	−8 × 10^4^	12	3.6	105	−8.3 × 10^−7^	7 × 10^−3^	−0.95
inh	−1.8 × 10^5^	16	3.0	120	1.67 × 10^−6^	6.2 × 10^−3^	4.17
exc,M	−3.2 × 10^5^	12	2	120^*^	−8.3 × 10^−7^	7 × 10^−3^	−0.95
inh,M	−3.5 × 10^5^	13.2	2	132^*^	1.67 × 10^−6^	6.2 × 10^−3^	4.17
**Additional parameters for monophasic filters**							
**Model (MOD)**				***T*_MOD, S_ (ms)**	***T*_MOD, C_ (ms)**	***R*_MOD_**	
exc,M				120	100	0.8	
inh,M				132	110	0.8	

Asterisks (^*^) indicate parameters that are superceded by later rows; note that the monophasic filter equations contain two filtering timescales—for example *T*_exc,M,S_ and *T*_exc,M,C_, for the excitatory monophasic filter—and a relative weighting (e.g., *R*_exc,M_).

For the filter demonstrated in Figure 4, we added a cosine component to the previous filter, i.e.,

(9) $$\begin{array}{l} L^{\text{exc,M}}(t)=P_{\text{exc,M}}{\left(t/\tau_{\text{exc,M}}\right)}^{n_{\text{exc,M}}}\exp\left(-t/\tau_{\text{exc,M}}\right)\\ \;\times \left[\sin\left(2\pi t/T_{\text{exc,M,S}}\right)+R_{\text{exc,M}}\cos\left(2\pi t/T_{\text{exc,M,C}}\right)\right] \end{array}$$

Here *P*_exc,M_ = −3.2 × 10^5^ s^−1^, *n*_exc,M_ = 2, τ_exc,M_ = 12 ms, *T*_exc,M,S_ = 120 ms and *T*_exc,M,C_ = 100 ms, and *P*_inh,M_ = −3.5 × 10^5^ s^−1^, *n*_inh,M_ = 2, τ_inh,M_ = 13.2 ms, *T*_inh,M,S_ = 132 ms and *T*_inh,M,C_ = 110 ms, while *R*_exc,M_ = *R*_inh,M_ = 0.8.

#### 4.1.2. Voltage evolution

We create a model of the cell as a non-linear integrate-and-fire model using the method of Badel et al. (2007), in which the membrane voltage is assumed to respond as

(10) $$\frac{dV}{dt}=F\left(V,t-t_{\text{last}}\right)+\frac{I_{\text{input}}(t)}{C}$$

where *C* is the cell capacitance, *t*_last_ is the time of the last spike before time *t*, and *I*_input_(*t*) is a time-dependent input current. We use the current-clamp data, which yields cell voltage in response to the input current *I*_input_(*t*) = −*g*^exc^(*t*)(*V* − *V*_*E*_) − *g*^inh^(*V* − *V*_*I*_), to fit a function *F*(*V, t*). When voltage data is segregated according to the time since the last spike *t* − *t*_last_, the *I* − *V* curve is well fit by a function of the form

(11) $$F\left(V,t-t_{\text{last}}\right)=\frac{1}{\tau_{m}}\left(E_{L}-V+\Delta_{T}e^{\left(V-V_{T}\right)/\Delta_{T}}\right)$$

where parameters are the membrane time constant τ_*m*_, resting potential (*E*_*L*_), spike width Δ_*T*_ and knee of the exponential curve *V*_*T*_.

The values of these constants differed in each bin of voltage data; to estimate these constants, we first extracted their values from each mean *I* − *V* curve. We found that these constants, as a function of *t* − *t*_last_, were well fit by either a single exponential or a difference of two exponentials, with relaxation to a baseline rate (as in Badel et al., 2007, Figure 3). Specifically, we chose:

(12) $$\begin{array}{l} \frac{1}{\tau_{m}}=c_{\tau_{m},1}+c_{\tau_{m},2}e^{-\left(t-t_{\text{last}}\right)/c_{\tau_{m},3}}\\ E_{L}=c_{E_{L},1}+c_{E_{L},2}\left(e^{-\left(t-t_{\text{last}}\right)/c_{E_{L},3}}-e^{-\left(t-t_{\text{last}}\right)/c_{E_{L},4}}\right)\\ \Delta_{T}=c_{\Delta_{T},1}+c_{\Delta_{T},2}\left(e^{-\left(t-t_{\text{last}}\right)/c_{\Delta_{T},3}}-e^{-\left(t-t_{\text{last}}\right)/c_{\Delta_{T},4}}\right)\\ V_{T}=c_{V_{T},1}+c_{V_{T},2}e^{-\left(t-t_{\text{last}}\right)/c_{V_{T},3}} \end{array}$$

We obtained the coefficients by least-squares fitting to the above functional forms: specifically, we found that (up to four digits): (*c*_τ_*m*_,1_, *c*_τ_*m*_,2_, *c*_τ_*m*_,3_) = (0.3719 ms^−1^, 0.5412 ms^−1^, 13.2726 ms), (*c*_*E*_*L*_,1_, *c*_*E*_*L*_,2_, *c*_*E*_*L*_,3_, *c*_*E*_*L*_,4_) = (−59.4858 mV, 5.8966 mV, 8.3076 ms, 233.1114 ms), (*c*_Δ_*T*_,1_, *c*_Δ_*T*_,2_, *c*_Δ_*T*_,3_, *c*_Δ_*T*_,4_) = (20.0487 ms, 19.0560 ms, 3.6280 ms, 2.4304 s), and (*c*_*V*_*T*_,1_, *c*_*V*_*T*_,2_, *c*_*V*_*T*_,3_) = (−44.3323 mV, 25.1812 mV, 4.7653 ms). Coefficients are also listed in Table 2.

**Table 2:** **Coefficients used to define refractory EIF model as specified in Equations (11, 12)**.

**Parameter (PAR)**	***c*_PAR,1_**	***c*_PAR,2_**	***c*_PAR,3_ (ms)**	***c*_PAR,4_ (ms)**
τ_*m*_ (actual fit: 1/τ_*m*_)	0.3719 ms^−1^	0.5412 ms^−1^	13.2726	
*V*_*T*_	−44.3323 mV	25.1812 mV	4.7653	
*E*_*L*_	−59.4858 mV	5.8966 mV	8.3076	233.1114
Δ_*T*_	20.0487 ms	19.0560 ms	3.6280	2430.4

The parameters *1*/τ_*m*_ and *V*_*T*_ were fit to single exponentials as functions of time, with three free parameters. The parameters *E*_*L*_ and Δ_*T*_ were fit to differences of exponentials and therefore have four parameters. Units in the first and second columns are as stated; coefficients in the third and fourth column are in units of milliseconds (ms).

The capacitance was inferred from the voltage trace data by finding, at a voltage value where the voltage/membrane current relationship is approximately Ohmic, the value of *C* that minimizes error in the relation Equation (10) (Badel et al., 2007). The estimated value was *C* = 28 pF.

#### 4.1.3. Spiking dynamics: feedforward network

For simulations without electronic coupling, our model neuron comprises Equations (10, 11) for *V* < *V*_threshold_; a spike was detected when *V* reached *V*_threshold_ = −30 mV; voltage was then reset to *V*_reset_ = −55 mV. The cell was then unable to spike for an absolute refractory period of τ_abs_ = 3 ms.

All simulations presented here were done in a three-cell network.

#### 4.1.4. Spiking dynamics: recurrent network

Gap junction coupling was introduced as an additional current on the right-hand side of Equation (10):

(13) Igap,jC=−ggapC∑k=j(Vj−Vk)

The coupling strength *g*^gap^ was held constant during a simulation. When coupling was present (i.e., when *g*^gap^ ≠ 0), *g*^gap^ was varied from the measured level (1.1 nS) (Trong and Rieke, 2008) to 16 times this value (17.6 nS) between simulations. When present, coupling was all-to-all.

As in the feedforward model, Equations (10, 11) were integrated for *V* < *V*_threshold_, and a spike was detected when *V* reached *V*_threshold_ = −30 mV. To model the voltage trajectory immediately following a spike, an averaged spike waveform was extracted from voltage traces of the same ON parasol cell used to fit Equations (10, 11). This spike waveform was then used to replace 1 ms of the membrane voltage trajectory during and after a spike; at the end of the 1 ms, the voltage was released at approximately −58 mV. The cell was unable to spike for an absolute refractory period of τ_abs_ = 3 ms. A relative refractory period was induced by introducing a declining threshold for the period of 3–6 ms following a spike, after which *V*_threshold_ returns to −30 mV.

#### 4.1.5. Cell receptive field and stimulation

We defined each cell's stimulus as the linear convolution of an image with its receptive field. The receptive fields include an ON center and an OFF surround, as in Chichilnisky and Kalmar (2002):

(14) $$\begin{array}{l} s_{j}\left(\vec{x}\right)=\exp\left(-\frac{1}{2}{\left(\vec{x}-{\vec{x}}_{j}\right)}^{T}Q\left(\vec{x}-{\vec{x}}_{j}\right)\right)\\ \;-k\exp\left(-\frac{1}{2}r\left(\vec{x}-{\vec{x}}_{j}\right)Qr\left(\vec{x}-{\vec{x}}_{j}\right)\right) \end{array}$$

where the parameters *k* and 1/*r* give the relative strength and size of the surround. **Q** specifies the shape of the center and was chosen to have a 1 standard deviation (SD) radius of 50 μm and to be perfectly circular. The receptive field locations $\vec{x}$_1_, $\vec{x}$_2_, and $\vec{x}$_3_ were chosen so that the 1 SD outlines of the receptive field centers will tile the plane (i.e., they just touch). Other parameters used were *k* = 0.3, *r* = 0.675.

Stimulation images were defined on a 512 μm × 512 μm grid that overlapped all three receptive fields. For full-field stimuli, light intensity was chosen be spatially constant and refreshed every 8, 40, or 100 ms by choosing independently from the specified stimulus distribution (Gaussian, binary, Cauchy, or heavy-tailed skew). For spatially variable stimuli, a checkerboard pattern was imposed on the stimulation image: the intensity value in each checkerboard square was chosen independently and refreshed at the appropriate interval. The checkerboard pattern was first given a random rotation and translation relative to the receptive fields: this was chosen at the outset of each batch of stixel simulations (for a total of five rotation/translation pairs per stixel size, refresh rate, and stimulus distribution). Two example placements are shown in Figures S2A,D for 256 μm and 60 μm pixels respectively.

#### 4.1.6. Numerical methods

All simulations and data analysis were performed using MATLAB. Equations (10, 11) were integrated using the Euler method for >10^5^ ms with a time step of 0.1 ms. The synaptic noise terms, η^exc^_*k*_ and η^inh^_*k*_, as well as the light input, were generated independently for each simulation. In response to uniform light stimuli, firing rates were 11.51 ± 0.38 Hz (standard deviations given across a total of 60 cells; 3 cells each from 20 10^5^ ms simulations); 10 ms bins were used to discretize the spiking output. Firing rates were higher for full-field stimuli, ranging from 12 to 43 Hz (firing rates increased with stimulus variance); therefore shorter (5 ms) bins were used to discretize spike output for all other simulations. With this range of firing rates and bin size, multiple spikes were very rare (occurring in <1% of occupied bins). Empirical spiking distributions were computed from the binned spike data.

For each stimulus condition, 20 simulations (or sub-simulations) were run, for a total integration time of > 20 × 10^5^ ms. These 20 sub-simulations were used to estimate standard errors in both the probability distribution over spiking events and *D*_KL_(*P*, $\tilde{P}$). Numbers reported in section 2 are, unless specified otherwise, produced by collating the data from the 20 simulations.

To fit a maximum entropy model $\tilde{P}$ to an empirical probability distribution *P*, we used standard methods that have been explained elsewhere (Malouf, 2002). Briefly, we minimized the negative log-likelihood function:

(15) $$L\left(\lambda \right)=-\sum_{x}P\left(x\right)\log\tilde{P}\left(x,\lambda \right)$$

where

$$\tilde{P}\left(x,\lambda \right)=Z_{\lambda }^{-1}\exp\left(\sum_{k}\lambda_{k}f_{k}\left(x\right)\right);$$

*Z*_λ_ is the partition function, *f*_*k*_, *k* = 1, …, *M* is a set of functions or “features” of the spiking state, and **λ** is a vector of parameters, each of which serves as a Lagrange multiplier enforcing the constraint **E**_$\tilde{P}$_[*f*_*k*_]. For the pairwise (PME) model on *N* cells, **λ** corresponds to *N* firing rates and *N*(*N* − 1)/2 covariances, and the sum is over all possible spiking states of the system. For *N* = 3 there are six such parameters, and

$$\begin{array}{l} \log\tilde{P}\left(\{x_{1},x_{2},x_{3}\},\lambda \right)=\lambda_{1}x_{1}+\lambda_{2}x_{2}+\lambda_{3}x_{3}+\lambda_{1,2}x_{1}x_{2}\\ \;+\lambda_{2,3}x_{2}x_{3}+\lambda_{1,3}x_{1}x_{3}-\log Z_{\lambda }. \end{array}$$

The function in Equation (15) is a convex function of the parameters **λ** which will be minimized precisely (and uniquely) when $\tilde{P}$ matches the desired moments from *P*: e.g., **E**_*P*_[*x*_1_] = **E**_$\tilde{P}$_[*x*_1_]. Since $\tilde{P}$ is in log-linear form, the result will be the *maximum entropy* distribution that matches the desired moments (Malouf, 2002). In principle any unconstrained gradient descent method may be used; we used an implementation of the non-linear conjugate gradient method. The Kullback Leibler divergence *D*_KL_(*P*, $\tilde{P}$) was computed using the identity *D*_KL_(*P*, $\tilde{P}$) = *S*($\tilde{P}$) − *S*(*P*), where *S*(*P*) is the entropy of *P*, i.e., *S*(*P*) = −∑_**x**_ *P*(**x**) log *P*(**x**).

#### 4.1.7. Convergence testing

To test our finding that the observed distributions were well-modeled by the PME fit, we also performed the PME analysis on each of the 20 simulations for each stimulus condition. While in general *D*_KL_(*P*, $\tilde{P}$) can be quite sensitive to perturbations in *P*, the numbers remained small under this analysis. To confirm that our results for *D*_KL_(*P*, $\tilde{P}$) are sufficiently resolved to remove bias from sampling, we performed an analysis in which we collect the 20 simulations in subgroups of 1, 2, 4, 5, 10, and 20, and plot the mean *D*_KL_ with estimated standard errors. As expected (e.g., Paninski, 2003), bias decreases as the length of subgroup increases and asymptotes at—or before—the full simulation length.

To provide a cross-validation test for the significance of our reported *D*_KL_(*P*, $\tilde{P}$) values, we divided our data into halves (which we denote *P*_1_ and *P*_2_, each including data from 10 sub-simulations) and performed the PME analysis on one half (say *P*_1_) to yield a model $\tilde{P}$_1_. We then computed *D*_KL_(*P*_2_, $\tilde{P}$_1_) and *D*_KL_(*P*_2_, *P*_1_) (as in Yu et al., 2011), which we refer to the *cross-validated* and *empirical* likelihood, respectively. The former tests whether the PME fit is robust to over-fitting; the latter tests how well-resolved our “true” distribution is in the first place. Most cross-validated likelihoods fall on or near the identity line; most empirical likelihoods are close to zero [and importantly, significantly smaller than either *D*_KL_(*P*, $\tilde{P}$) or *D*_KL_(*P*_2_, $\tilde{P}$_1_), indicating that *D*_KL_(*P*, $\tilde{P}$) is accurately resolved]. We conclude that the deviations that we observe when these conditions are met can not be accounted for by the differences in testing and training data.

### 4.2. Computation of spiking patterns in the simplified model

As a simplified model of a neural circuit, we consider a variant of the *Dichotomized Gaussian* (Amari et al., 2003; Macke et al., 2009, 2011), in which correlated inputs are thresholded to produce an output spike pattern. To be concrete, a set of *N* threshold spiking units is forced by a common input *I*_*c*_ [drawn from a probability distribution *P*_*C*_(*y*)] and an independent input *I*_*j*_ [drawn from a probability distribution *P*_*I*_(*y*)]. To relate these functions to the other free parameters in the model, *P*_*C*_(*y*) and *P*_*I*_(*y*) were always chosen so that *I*_*j*_ and *I*_*c*_ had mean 0 and variances (1 − *c*) σ^2^ and *c* σ^2^, respectively (so that *c* yields the Pearson's correlation coefficient of the input to two cells). The output of each cell *x*_*j*_ is determined by summing and thresholding these inputs:

(16) $$x_{j}=H\left(I_{j}+I_{c}-\Theta \right)$$

where *H* is the Heaviside function [*H*(*x*) = 1 if *x* ≥ 0; *H*(*x*) = 0 otherwise]. Conditioned on *I*_*c*_, the probability of each spike is given by:

$$\begin{array}{l} Prob\left[x_{j}=1|I_{c}=a\right]=Prob\left[I_{j}+a-\Theta >0\right]\\ \;=Prob\left[I_{j}>\Theta -a\right]\\ \;=\int_{\Theta -a}^{\infty }P_{I}(y)dy \end{array}$$

Similarly, we have the conditioned probability that *x*_*j*_ = 0:

$$\begin{array}{l} Prob\left[x_{j}=0|I_{c}=a\right]=Prob\left[I_{j}+a-\Theta <0\right]\\ \;=Prob\left[I_{j}<\Theta -a\right]\\ \;=\int_{-\infty }^{\Theta -a}P_{I}(y)dy \end{array}$$

Because these are conditionally independent, the probability of any spiking event (*x*_1_, *x*_2_, …, *x*_*N*_) = (*A*_1_, *A*_2_, …, *A*_*N*_) is given by the integral of the product of the conditioned probabilities against the density of the common input.

(17) $$\begin{array}{l} Prob\left[x_{1}=A_{1},\ldots ,x_{N}=A_{N}\right]=\int_{-\infty }^{\infty }dyP_{C}(y)\\ \;\prod_{j=1}^{N}Prob\left[x_{j}=A_{j}|I_{c}=y\right] \end{array}$$

The integral in Equation (17) is numerically evaluated via an adaptive quadrature routine, such at Matlab's quad or integral.

Four distinct unimodal inputs were used; two with heavy tails (Cauchy and heavy-tailed with skew), and two with sub-Gaussian tails (Gaussian and skewed). A random variable *X* is *sub-Gaussian* if the probability of large events can be bounded above by a scaled Gaussian; that is, if there exist constants *C*, *c* > 0 such that

$$P\left(|X|>\lambda \right)\leq C\exp\left(-c\lambda^{2}\right)$$

for all λ (e.g., see Tao, 2012, p. 15).

Unimodal inputs *I*_*j*_, *I*_*c*_ were chosen from different marginals with mean 0 and variances (1 − *c*) σ^2^, *c* σ^2^, respectively (for simplicity, we use σ^2^ to refer to the variance of a generic probability distribution in the following three paragraphs). For Gaussian inputs with variance σ^2^, *P*(*x*) ∝ *e*^−*x*^2^/2σ^2^^; for skewed inputs, *P*(*x*) ∝ (*x* + μ)*e*^−(*x* + μ)^2^/2*a*^, for *x* > −μ, where the parameter *a* sets the variance $2a(1-\frac{\pi }{4})$ and shifting by $\mu =\sqrt{\frac{a\pi }{2}}$ ensures that the mean of *P*(*x*) is zero.

The heavy-tailed unimodal inputs were chosen so that the rate of tail decay would mimic the *I*^−2^ luminance statistics found in natural scenes (Ruderman and Bialek, 1994):

$$\begin{array}{l} \;P(x)\propto \frac{1}{x^{2}+1},\;-X<x<X\\ P(x)\propto \frac{x}{{\left(x^{2}+1\right)}^{3/2}},\;0\leq x<X \end{array}$$

Bimodal inputs with variance σ^2^ were chosen in the following way: in all cases, *P*(*x*) was chosen to be a discrete distribution with support on two values {0, X} i.e., *P*(*X*) = *p* and *P*(0) = 1 − *p*. If possible (i.e., if σ^2^ ≤ 1/4), *X* was chosen to be 1; otherwise, *X* was chosen so as to minimize the distance between 0 and *X*. Finally, *P*(*x*) was shifted to have the desired mean value.

### Conflict of interest statement

The authors declare that the research was conducted in the absence of any commercial or financial relationships that could be construed as a potential conflict of interest.

## Supplementary material

Figure S1 **Biphasic vs. monophasic filters used in simulations illustrated in Figure 4**.

Figure S2 **Illustration of RGC simulations with light stimuli of varying spatial scale (“stixels”)**. **(A–C)** For stixel size 60 μm, results for one randomly chosen stimulus position. **(A)** Contour lines of the three receptive fields (at 0.5, 1, 1.5, and 2 SD; and at the zero contour line) superimposed on the stimulus checkerboard (for illustration, pictured in an alternating black/white pattern). The red scale bar indicates 100 μm. **(B)** Histograms of the excitatory conductances, for each cell. **(C)** Spike pattern distribution, as obtained from computational simulations of the RGC model (“Observed”; dark blue), and the corresponding pairwise fit (“PME”; light pink). All eight spike patterns are shown, to allow for the possibility of non-symmetric responses; the three different probabilities labeled *p*_1_ correspond to *P*[(1, 0, 0)], *P*[(0, 1, 0)], and *P*[(0, 0, 1)]. **(D–F)** As in **(A–C)**, but for stixel size 256 μm. Panels **(E,F)** demonstrate that for this input, both excitatory inputs and spiking responses are heterogenous across the RGCs.

Figure S3 **Strength of higher-order interactions produced by the threshold model as input parameters vary; relationship with other output firing statistics**. **(A)** For skewed common inputs: *D*_KL_(*P*, $\tilde{P}$) as a function of input correlation *c* and input standard deviation σ, for a fixed threshold Θ = 1.5. Color indicates *D*_KL_(*P*, $\tilde{P}$); see color bar for range. **(B)** For skewed common inputs: *D*_KL_(*P*, $\tilde{P}$) vs. firing rate **E**[*x*_1_] (Left) and the fraction of multi-information (Δ) captured by the PME model vs. firing rate **E**[*x*_1_] (Right). In **(B)**, possible input parameters were varied over a broad range as described in section 2. Firing rate is defined as the probability of a spike occurring per cell per random draw of the sum-and-threshold model, as defined in Equation (16). Color indicates output correlation coefficient ρ ranging from black for ρ ∈ (0, 0.1), to white for ρ ∈ (0.9, 1), as illustrated in the color bars. **(C,D)**: as in **(A,B)**, but for heavy-tailed, skewed common inputs.

Figure S4 **The range of higher-order interactions produced by the threshold model varies across input type**. Here, all values of *D*_KL_(*P*, $\tilde{P}$) produced by the three-cell threshold model (previously displayed in Figures 7, S3) are superimposed to show the contrast between different input distributions. By comparing these data with data from direct sampling of all symmetric spiking distributions on three cells (from Figure 1 and shown here in yellow), one can see that only a limited set of output patterns are accessed by the feedforward thresholding model. Firing rate is defined as the probability of a spike occurring per cell per random draw of the sum-and-threshold model, as defined in Equation (16).

## Appendix

### A.1. A measure of higher-order interactions: *D*_*KL*_(*P*, $\tilde{P}$)

We begin by observing that when $\tilde{P}$ is a maximum entropy distribution that approximates *P* (that is, it is log-linear, with coefficients chosen to enforce equality of a set of moments), then the KL-distance may be written as a difference of entropies (Cover and Thomas, 1991; Malouf, 2002):

$$D_{\text{KL}}\left(P,\tilde{P}\right)=-S(P)+S\left(\tilde{P}\right)$$

Here, the entropy of a probability distribution *P* on {0, 1}^3^ is given

(18) $$\begin{array}{l} S\text{​}\left(P\right)=-p_{0}\log\left(p_{0}\right)-3p_{1}\log\left(p_{1}\right)-3p_{2}\log\left(p_{2}\right)\\ \;-p_{3}\log\left(p_{3}\right) \end{array}$$

if we use the fact that the distributions are permutation-symmetric [i.e., *p*_1_ ≡ *P*(1, 0, 0) = *P*(0, 1, 0) = *P*(0, 0, 1)]. We take the logarithms in the definitions of the entropy *S* and KL-divergence *D*_KL_ to be base 2, so that any numerical values of these quantities are in units of bits. Using the fact that *P* must normalize to 1, we rewrite

$$\begin{array}{l} S\text{​}\left(P\right)=-\left(1-3p_{1}-3p_{2}-p_{3}\right)\log\left(1-3p_{1}-3p_{2}-p_{3}\right)\\ \;-3p_{1}\log\left(p_{1}\right)-3p_{2}\log\left(p_{2}\right)-p_{3}\log\left(p_{3}\right) \end{array}$$

where the set of admissible distributions may now be described by the convex tetrahedron in ℝ^3^,  = {*p*_1_, *p*_2_, *p*_3_ ≥ 0; 3*p*_1_ + 3*p*_2_ + *p*_3_ ≤ 1}

We note that the set of distributions which satisfies a desired set of lower order moments is given by an affine subspace (in ℝ^3^, a line) which intersects this tetrahedron:

$$\begin{array}{l} \mu \equiv E[X_{i}]=p_{1}+2p_{2}+p_{3}\\ \hat{\rho }\equiv E[X_{i}^{2}]=p_{2}+p_{3} \end{array}$$

Denoting this set by _μ, $\hat{\rho }$_, we note that _μ, $\hat{\rho }$_ is a convex set and that *S*($\tilde{P}$) is constant on each _μ, $\hat{\rho }$_.

By straightforward differentiation we can check that the Hessian of −*S*(*P*) is positive definite, as long as the probabilities *p*_0_, *p*_1_, etc. are strictly greater than zero:

$$-D^{2}S(P)=\left[\begin{array}{ccc} \frac{3}{p_{1}} & 0 & 0\\ 0 & \frac{3}{p_{2}} & 0\\ 0 & 0 & \frac{1}{p_{3}} \end{array}\right]+\frac{1}{p_{0}}\left[\begin{array}{ccc} 9 & 9 & 3\\ 9 & 9 & 3\\ 3 & 3 & 1 \end{array}\right]$$

Therefore −*S*(*P*) is convex on _μ, $\hat{\rho }$_; since *S*($\tilde{P}$) is constant, *D*_KL_(*P*, $\tilde{P}$) is likewise convex on _μ, $\hat{\rho }$_. As a consequence, if *D*_KL_(*P*, $\tilde{P}$) has a local minimum, then it is unique and a global minimum as well. Since *D*_KL_(*P*, $\tilde{P}$) ≥ 0 with equality if and only if *P* = $\tilde{P}$, this minimum must be achieved occurs when *P* = $\tilde{P}$; the maximum is likewise achieved on the boundary of the admissible region _μ, $\hat{\rho }$_.

### A.2. A measure of higher-order interactions: strain

We define the *strain*,

(19) $$\begin{array}{l} \gamma =\log\left(\frac{p_{3}p_{1}^{3}}{p_{0}p_{2}^{3}}\right)\\ \;=\log p_{3}-\log p_{0}+3\log p_{1}-3\log p_{2} \end{array}$$

a potential measure of the importance of higher-order interactions (Ohiorhenuan and Victor, 2010). By Equation (3), we can see that γ = 0 precisely for a pairwise maximum entropy (PME) distribution. We will show that as the distribution (*p*_0_, *p*_1_, *p*_2_, *p*_3_) is moved away from the constraint surface while fixing lower-order moments, the strain increases monotonically.

From the definition of lower-order moments,

$$\begin{array}{l} \mu =E[X_{i}]=p_{1}+2p_{2}+p_{3}\\ \hat{\rho }=E[X_{i}X_{j}]=p_{2}+p_{3} \end{array}$$

we can verify that in order to keep μ, $\hat{\rho }$ constant, if *p*_1_ increases by *z* (i.e., *p*_1_ → *p*_1_ + *z*), then we must also have *p*_2_ → *p*_2_ − *z* and *p*_3_ → *p*_3_ + *z*. Then if each probability is strictly positive, then the derivative

$$\frac{\partial \gamma }{\partial z}=\frac{1}{p_{3}+z}+\frac{1}{1-p_{3}-3p_{1}-3p_{2}-z}+\frac{3}{p_{1}+z}+\frac{3}{p_{2}-z}$$

is strictly positive as well. In particular, it is strictly positive at *z* = 0 and will remain positive until *z* reaches a value such that one of the denominators reaches 0. Therefore γ increases monotonically for *z* > 0 and decreases monotonically for *z* < 0.

### A.3. An analytical explanation for unimodal vs. bimodal effects

We consider an analytical argument to support the numerical results that bimodal inputs generate larger deviations from PME model fits than unimodal inputs. As a metric, we consider *D*_KL_(*P*, $\tilde{P}$)—where *P* and $\tilde{P}$ are again the true and model distributions, respectively—when we perturb an independent spiking distribution by adding a common, global input of variance *c*. To simplify notation, the small parameter in the calculation will be denoted $\epsilon =\sqrt{c}$.

We now compute *S*(*P*) and *S*($\tilde{P}$) (defined in an earlier Appendix) by deriving a series expansion for each set of event probabilities. We can compute the true distribution *P* using the expressions derived in Equation (18); to recap, let the common input *I*_*c*_ have probability density *p*(*I*_*c*_), and the independent input to each cell, *x*, have density *p*_*s*_(*x*). Let Θ be the threshold for generating a spike (i.e., a “1” response). For each cell, a spike is generated if *x* + *I*_*c*_ > Θ, i.e., with probability

$$d(I_{c})=\int_{\Theta -I_{c}}^{\infty }p_{s}(x)dx.$$

Given *I*_*c*_, this is conditionally independent for each cell. We can therefore write our probabilities by integrating over *I*_*c*_ as follows:

(20) $$\begin{array}{l} p_{0}=\int_{-\infty }^{\infty }p\left(I_{c}\right){\left(1-d\left(I_{c}\right)\right)}^{3}dI_{c}\\ p_{1}=\int_{-\infty }^{\infty }p\left(I_{c}\right)d\left(I_{c}\right){\left(1-d\left(I_{c}\right)\right)}^{2}dI_{c}\\ p_{2}=\int_{-\infty }^{\infty }p\left(I_{c}\right)d{\left(I_{c}\right)}^{2}\left(1-d\left(I_{c}\right)\right)dI_{c}\\ p_{3}=\int_{-\infty }^{\infty }p\left(I_{c}\right)d{\left(I_{c}\right)}^{3}dI_{c} \end{array}$$

We develop a perturbation argument in the limit of very weak common input. That is, *p*(*I*_*c*_) is close to a delta function centered at *I*_*c*_ = 0. Take *p*(*I*_*c*_) to be a scaled function

(21) $$p(I_{c})=\frac{1}{\epsilon }f\left(\frac{I_{c}}{\epsilon }\right)$$

We place no constraints on *f*(*x*), other than that it must be normalized (**E**[1] = 1) and that its moments must be finite (so that **E**[*I*_*c*_], **E**[*I*^2^_*c*_], and so forth will exist, where **E**[*g*(*x*)] ≡ ∫^∞^_−∞_ *g*(*x*) *f*(*x*) *dx*).

For the moment, assume that the function *f*(*x*) has a single maximum at *x* = 0. To evaluate the integrals above, we Taylor-expand *d*(*x*) around *x* = 0. Anticipating a sixth-order term to survive, we keep all terms up to this order. This gives, for small *x*,

$$d(x)\approx d(0)+\sum_{k=1}^{6}a_{k}x^{k}+O(x^{7})$$

where *a*_1_ = *p*_*s*_(Θ) (the other coefficients *a*_2_ − *a*_6_ can be given similarly in terms of the independent input distribution at Θ). Substituting this into the expressions for *p*_0_, etc., above, with *p*(*I*_*c*_) given as in Equation (21), gives us each event as a series in ϵ; for example,

$$p_{3}=d_{0}^{3}+\left(3a_{1}d_{0}^{2}E[x]\right)\epsilon +\left(\left(3a_{1}^{2}d_{0}+3a_{2}d_{0}^{2})E[x^{2}\right]\right)\epsilon^{2}+\ldots ,$$

where expectations are, again, with respect to the unscaled PDF *f*(*x*). The entropy *S*(*P*) is now given by using these series expansions in Equation (18).

We note that our derivation does not rely on the fact that the distribution of common input is peaked at *I*_*c*_ = 0 in particular. For example, we could have a common input centered around μ. The common input distribution function would be of the form

$$p(I_{c})=\frac{1}{\epsilon }f\left(\frac{I_{c}-\mu }{\epsilon }\right)$$

Changing ϵ regulates the variance, but doesn't change the mean or the peak (assuming, without loss of generality, that the peak of *f* occurs at zero). The peak of *p*(*I*_*c*_) now occurs at μ, and the appropriate Taylor expansion of *d*(*x*) is

$$d(x)\approx d(\mu )+\sum_{k=1}^{6}b_{k}{\left(x-\mu \right)}^{k}+O(x^{7}),$$

where the coefficients *b*_*k*_ now depend on the local behavior of *d* around μ. The expectations that appear in the expansion of *p*_3_, and so forth, are now centered moments taken around μ; the calculations are otherwise identical. In other words, the perturbation expansion requires the *variance* of the common input to be small, but not the mean.

For bimodal inputs, we consider a common input with a probability distribution of the following form:

$$p\left(I_{c}\right)=\left(1-\epsilon^{2}\right)\frac{1}{\epsilon }f\left(\frac{I_{c}}{\epsilon }\right)+\epsilon^{2}\frac{1}{\epsilon }f\left(\frac{I_{c}-1}{\epsilon }\right)$$

so that most of the probability distribution is peaked at zero, but there is a second peak of higher order (here taken at *I*_*c*_ = 1, without loss of generality). Again, we approximate the integrals given in Equation (20), and therefore the entropy *S*(*P*), by Taylor expanding *d*(*x*);

$$\begin{array}{l} d(x)\approx d(0)+\sum_{k=1}^{6}a_{k}x^{k}+O(x^{7});\;\left(x\approx 0\right)\\ \;\approx d(1)+\sum_{k=1}^{6}b_{k}{\left(x-1\right)}^{k}+O\left({\left(x-1\right)}^{7}\right);\;\left(x\approx 1\right) \end{array}$$

around the two peaks 0 and 1, respectively. For each integral we have the same contributions from the unimodal case, multiplied by (1 − ϵ^2^), as well as the corresponding contributions from the second peak multiplied by ϵ^2^ (these weightings are chosen so that the common input has variance of order ϵ^2^, as in the unimodal case). This makes clear at what order every term enters.

We now construct an expansion for the PME model $\tilde{P}$:

$$\begin{array}{l} \tilde{P}\left(x_{1},x_{2},x_{3}\right)=\frac{1}{Z}\exp(\lambda_{1}\left(x_{1}+x_{2}+x_{3}\right)\\ \;+\lambda_{2}\left(x_{1}x_{2}+x_{2}x_{3}+x_{1}x_{3}\right)) \end{array}$$

We approach this problem by describing λ_1_ and λ_2_ as a series in ϵ. We match coefficients by forcing the first and second moments of $\tilde{P}$ to match those of *P*—as they must. Specifically, take

$$\begin{array}{l} \lambda_{1}=\tilde{\lambda }+\sum_{k=1}^{6}\epsilon^{k}u_{k}+O\left(\epsilon^{7}\right)\\ \lambda_{2}=\sum_{k=1}^{6}\epsilon^{k}v_{k}+O\left(\epsilon^{7}\right) \end{array}$$

where $\lambda_{1}=\tilde{\lambda }$, λ_2_ = 0 are the corresponding parameters from the independent case. The events $\tilde{p}$_0_, $\tilde{p}$_1_, $\tilde{p}$_2_, and $\tilde{p}$_3_ can be written as a series in ϵ. We then require that the mean and centered second moments of $\tilde{P}$ match those of *P*; that is

$$\begin{array}{l} \;p_{1}+2p_{2}+p_{3}={\tilde{p}}_{1}+2{\tilde{p}}_{2}+{\tilde{p}}_{3}\\ p_{2}+p_{3}-{\left(p_{1}+2p_{2}+p_{3}\right)}^{2}={\tilde{p}}_{2}+{\tilde{p}}_{3}-{\left({\tilde{p}}_{1}+2{\tilde{p}}_{2}+{\tilde{p}}_{3}\right)}^{2}. \end{array}$$

At each order *k*, this yields a system of two linear equations in *u*_*k*_ and *v*_*k*_; we solve, inductively, up to the desired order; we now have $\tilde{P}$, and therefore *S*($\tilde{P}$), as a series in ϵ.

Finally, we combine the two series to find that in the *unimodal* case,

(22) $$\begin{array}{l} D_{\text{KL}}\left(P,\tilde{P}\right)=S\left(\tilde{P}\right)-S(P)\\ \;=\epsilon^{6}\left[\frac{a_{1}^{6}{\left(2E{\left[x\right]}^{3}-3E[x]E[x^{2}]+E[x^{3}]\right)}^{2}}{2{\left(1-d_{0}\right)}^{3}d_{0}^{3}}\right]\\ \;+O\left(\epsilon^{7}\right) \end{array}$$

If the first two odd moments of the distribution are zero (something we can expect for “symmetric” distributions, such as a Gaussian), then this sixth-order term is zero as well.

For the *bimodal* case

$$\begin{array}{l} D_{\text{KL}}\left(P,\tilde{P}\right)=S\left(\tilde{P}\right)-S(P)\\ \;=\epsilon^{4}\left[\frac{{\left(d_{1}-d_{0}\right)}^{6}}{2{\left(1-d_{0}\right)}^{3}d_{0}^{3}}\right]+O\left(\epsilon^{5}\right) \end{array}$$

This last term depends on the distance *d*_1_ − *d*_0_, in other words, how much more likely the independent input is to push the cell over threshold when common input is “ON”. We can also view this as depending on the ratio $\frac{d_{1}-d_{0}}{1-d_{0}}$, which gives the fraction of previously non-spiking cells that now spike as a result of the common input.

*The main point here, of course, is that *D*_KL_(*P*, $\tilde{P}$) is of order ϵ^4^ rather than* ϵ^6^. So, as the strength of a common binary vs. unimodal input increases, spiking distributions depart from the PME more rapidly.
