# Supplementary figures and images for: When do microcircuits produce beyond-pairwise correlations?
Source: Front Comput Neurosci. 2014 Feb 6;8:10. doi: 10.3389/fncom.2014.00010 (PMC3915758; doi:10.3389/fncom.2014.00010)

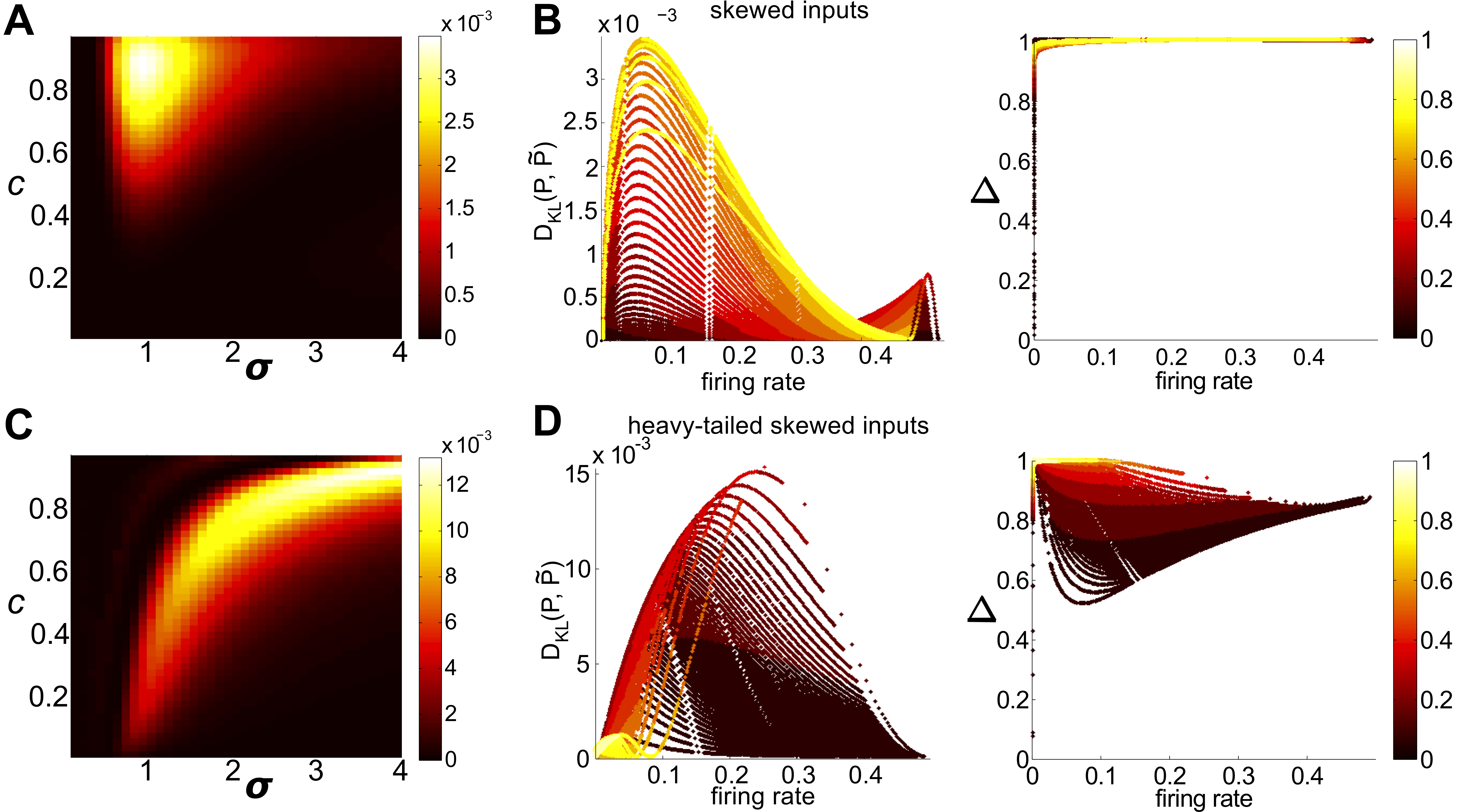

Supplement: Figure S1 — Biphasic vs. monophasic filters used in simulations illustrated in Figure 4. [file Presentation1.ZIP › 61336_Barreiro_Suppl_Figure_3.TIFF]

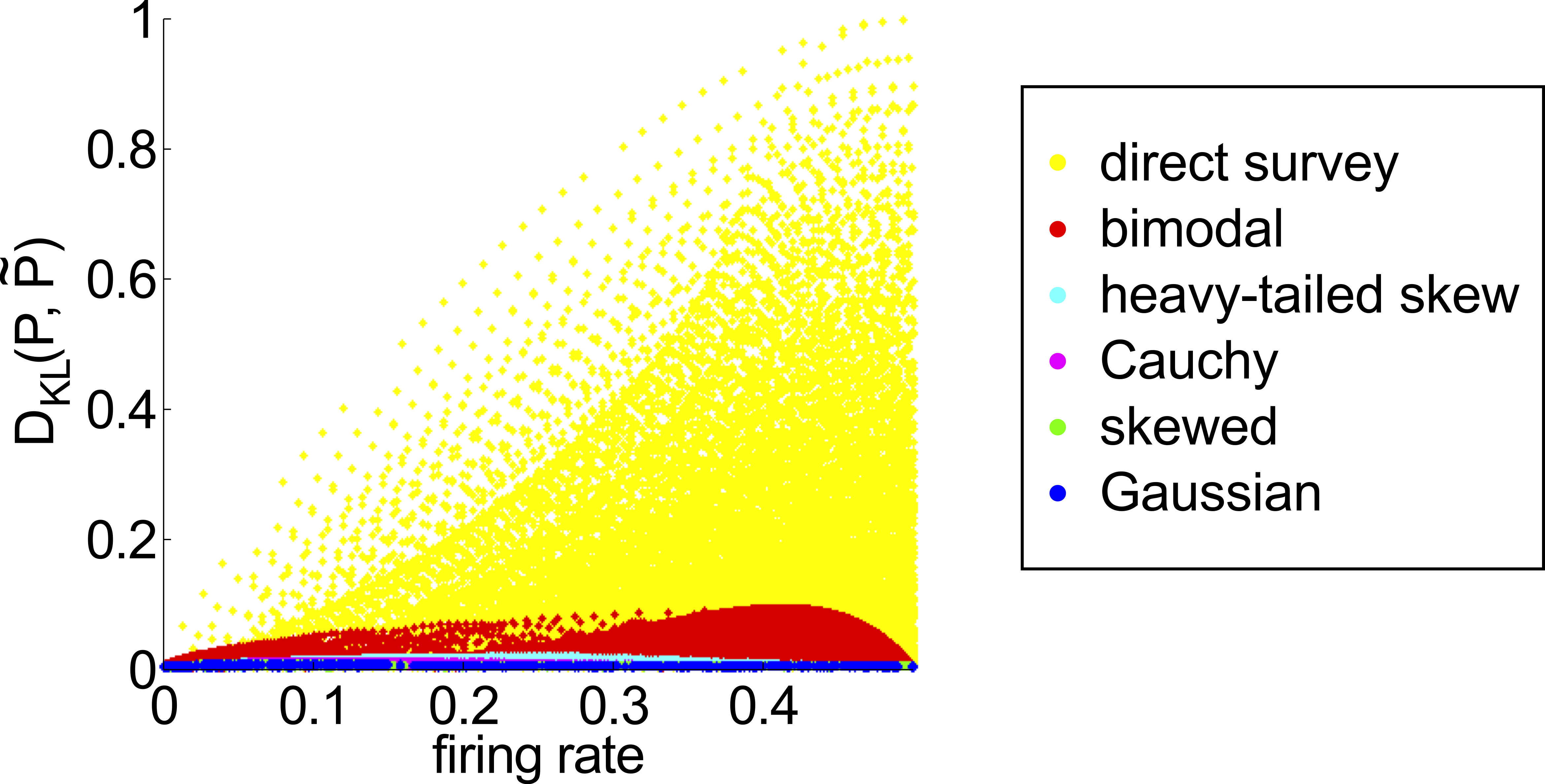

Supplement: Figure S1 — Biphasic vs. monophasic filters used in simulations illustrated in Figure 4. [file Presentation1.ZIP › 61336_Barreiro_Suppl_Figure_4.TIFF]

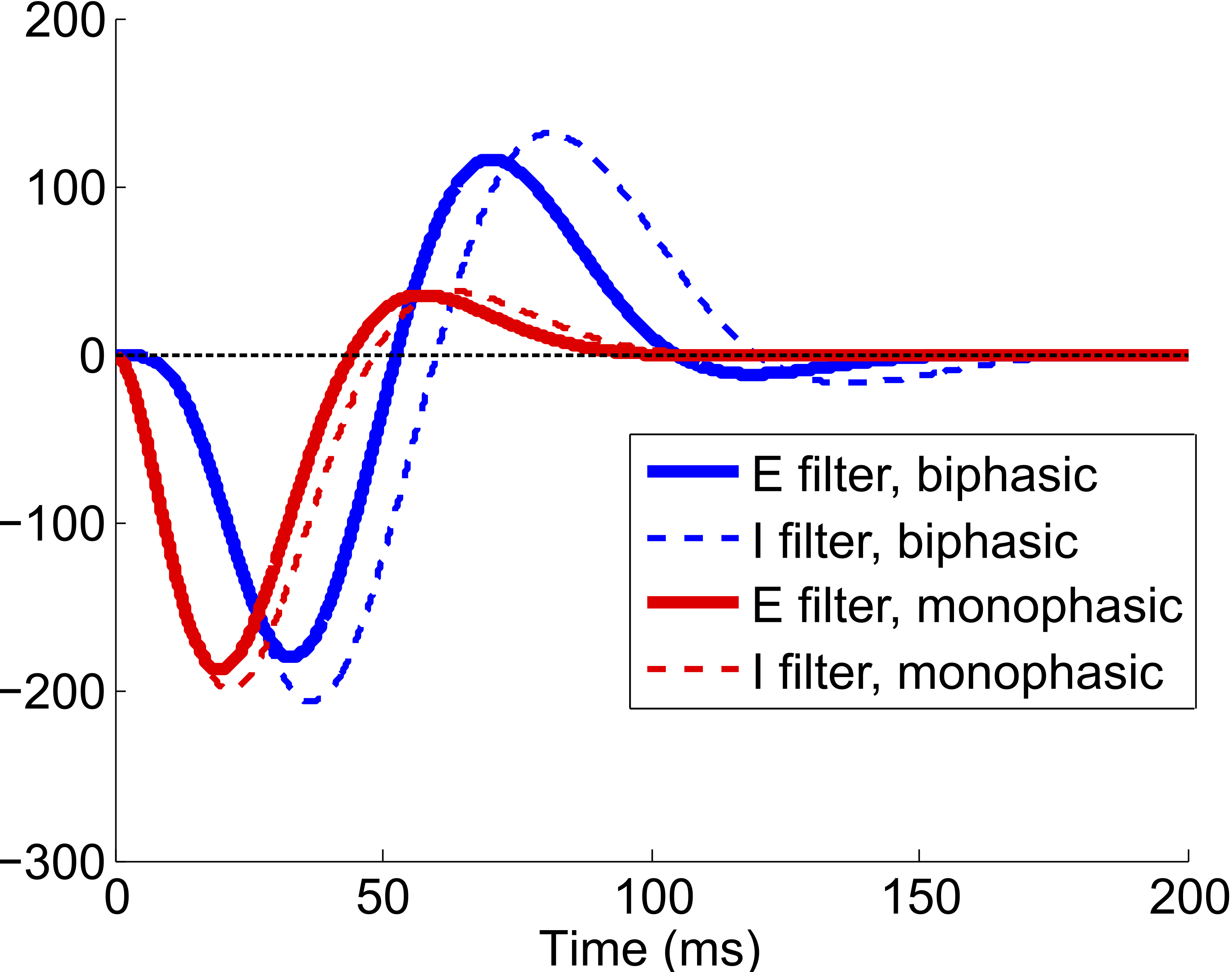

Supplement: Figure S1 — Biphasic vs. monophasic filters used in simulations illustrated in Figure 4. [file Presentation1.ZIP › 61336_Barreiro_Suppl_Figure_1.TIFF]
